# *Cryptococcus neoformans* Mediator Protein Ssn8 Negatively Regulates Diverse Physiological Processes and Is Required for Virulence

**DOI:** 10.1371/journal.pone.0019162

**Published:** 2011-04-29

**Authors:** Lin-Ing Wang, Yu-Sheng Lin, Kung-Hung Liu, Ambrose Y. Jong, Wei-Chiang Shen

**Affiliations:** 1 Department of Plant Pathology and Microbiology, National Taiwan University, Taipei, Taiwan; 2 Division of Hematology-Oncology, Childrens Hospital Los Angeles, Los Angeles, California, United States of America; University of Minnesota, United States of America

## Abstract

*Cryptococcus neoformans* is a ubiquitously distributed human pathogen. It is also a model system for studying fungal virulence, physiology and differentiation. Light is known to inhibit sexual development via the evolutionarily conserved white collar proteins in *C. neoformans*. To dissect molecular mechanisms regulating this process, we have identified the *SSN8* gene whose mutation suppresses the light-dependent *CWC1* overexpression phenotype. Characterization of sex-related phenotypes revealed that Ssn8 functions as a negative regulator in both heterothallic **a**-α mating and same-sex mating processes. In addition, Ssn8 is involved in the suppression of other physiological processes including invasive growth, and production of capsule and melanin. Interestingly, Ssn8 is also required for the maintenance of cell wall integrity and virulence. Our gene expression studies confirmed that deletion of *SSN8* results in de-repression of genes involved in sexual development and melanization. Epistatic and yeast two hybrid studies suggest that *C. neoformans* Ssn8 plays critical roles downstream of the Cpk1 MAPK cascade and Ste12 and possibly resides at one of the major branches downstream of the Cwc complex in the light-mediated sexual development pathway. Taken together, our studies demonstrate that the conserved Mediator protein Ssn8 functions as a global regulator which negatively regulates diverse physiological and developmental processes and is required for virulence in *C. neoformans*.

## Introduction

Transcription is an essential process for gene expression in both prokaryotic and eukaryotic organisms, which requires RNA polymerase to decode the DNA sequences. Transcription in eukaryotic cells is a complex process which requires the interactions of the RNA polymerase II (RNAPII) general transcriptional machinery with a whole array of DNA-binding transcriptional factors [1]. Activation and repression of RNAPII activity by the positive and negative regulatory proteins is fundamental to the regulation. An important group of highly conserved proteins forms the Mediator complex that functions in conveying the regulatory signals from these transcription factors to RNAPII [2]–[4]. The Mediator complex is comprised of 24 subunits in *Saccharomyces cerevisiae*. The number of subunits varies in different species, but overall, the Mediator is well conserved from yeast to human in its protein structure [5]–[7].

The Mediator subunits are organized structurally into 4 major parts: head, middle, tail and a free module [8]. The former three parts form the core Mediator which functions together with RNAPII as the holoenzyme [4]. Ssn8, also known as Srb11, a C-type Cyclin, and its specific Cyclin-dependent kinase (CDK), Ssn3/Srb10, are members of the free module in the Mediator complex. Therefore, this free module has a kinase role. The free module is recruited by repressors to phosphorylate Ser2 and/or Ser5 in the heptapeptide repeats of CTD [9], [10]. This phosphorylation of RNAP II complex, that prevents its interaction with the core Mediator, results in the repression of gene expression. On the other hand, transcription is activated when activators recruit the core Mediator complex without the free module to associate with RNAP II complex at the promoter region [2], [11], [12]. Although most studies indicate that the free module serves in a negative role, some have demonstrated its activation function. In *S. cerevisiae*, the free module is required for the induction of the *GAL* genes by phosphorylating the Gal4 activator which may prevent the repressor, Gal80, from binding to the promoter region [13], [14].

Ssn8 is involved in the regulation of carbon utilization in *S. cerevisiae*. It was first identified as a suppressor of the *snf1* mutation [15]. *SSN8* was later rediscovered as a suppressor in the *snf1mig1* double mutant screening for gluconeogenic growth [16]. Snf1 and Mig1 in yeast are two key regulators involved in carbon catabolite repression [17]. Ssn8, also named Ume3, plays critical roles in meiosis [18]. Destruction of Ssn8 is required for induction and execution of meiotic development; deletion of *SSN8* results in unscheduled meiotic gene expression [19]. Genome-wide studies in *S. cerevisiae* revealed that 173 genes (approximately 3% of the genome) are negatively regulated by the Srb11 containing free module, of these genes 75 genes are involved in nutrient scavenging and morphological change in response to nutritional stress [20]. Stress responses such as ethanol shock, heat shock and oxidative stress are regulated by *S. cerevisiae SSN*8 [21]–[23]. Being targeted at different domains, Ssn8 is degraded under different stress conditions and the responsive genes for particular stress are de-repressed [23]. For example under oxidative stress, the level of Ssn8 is under the regulation of Slt2 and Ask10, two downstream components of the PKC pathway [22], [24]. All these studies demonstrate that Ssn8 plays a global and vital role in yeast physiology.

In filamentous fungi, the *SSN8* homologues of *Fusarium verticillioides* and *F. graminearum*, *FCC1* and *CID1* respectively, have been identified and characterized. Deletion of the *SSN8* homologues in *Fusarium* spp. causes pleiotropic phenotypes. In *F. verticillioides*, a mutation of *FCC1* or *FCK1* shows reduced conidiation and fumonisin production on cultures at pH 6 and these two proteins have been shown to physically interact [25], [26]. In *F. graminearum*, *ssn8* mutant shows slow growth, reduced conidiation, increased pigmentation, female sterility and sensitivity to stress conditions. In addition, it also reduces the production of DON (deoxynivalenol), a protein synthesis inhibitor which causes toxicosis, and fails to infect corn plants [27]. These reports describe the global regulatory roles of *SSN8* homologues in filamentous fungi including plant fungal pathogens.

*Cryptococcus neoformans* is a globally distributed human fungal pathogen that exists in different ecological niches [28]. To adapt different environments and to adjust to the transition and challenges upon entering the host, various sensing mechanisms to co-opt the external cues or stimuli have been developed [29]. *C. neoformans* grows vegetatively as the yeast form; filamentation is primarily associated with two sexual processes, heterothallic **a**-α mating and α-α same-sex mating [30], [31]. Blue light is known to inhibit the production of sexual filaments in *C. neoformans* and two evolutionally conserved blue light regulators, Cwc1 and Cwc2, play critical roles in these processes [32], [33]. Mutants carrying a deletion in either *CWC1* or *CWC2* are blind to the inhibitory effect of light on mating and are sensitive to ultraviolet light. Interestingly, they also show a reduction of virulence in a murine model [32]. Elevating transcript level of *CWC1* or *CWC2* with synthetic constructs causes inhibition of mating filamentation in the light [33].

In order to dissect the molecular mechanisms of blue light-inhibited sexual development in *C. neoformans*, a genome wide mutagenesis was conducted under the *CWC1* overexpression background to screen for mutants that restored filamentous growth in the light [34]. A T-DNA insertion into the Mediator *SSN8* gene not only suppresses the light-dependent *CWC1* overexpression mating phenotype but also shows dramatic de-repression of same-sex mating [34]. In this study, we aimed to characterize the roles of Ssn8 in *C. neoformans* and its relationship to the light-mediated filamentation pathway. An *ssn8* mutation was introduced into different strain backgrounds and their phenotypic characterization was conducted under various conditions. The results indicate that *C. neoformans* Ssn8 functions as a global negative regulator involved in diverse physiological and developmental processes. In addition to negatively regulating filamentation in sexual development, Ssn8 also suppresses melanization, capsule formation and invasive growth, and is also required for the maintenance of cell wall integrity and virulence.

## Results

### *C. neoformans* Ssn8 Is a Mediator Protein Containing the Conserved Cyclin Box and PEST Domain

Our previous studies showed that *C. neoformans* Cwc1 and Cwc2 are two central regulators which coordinately mediate blue light-inhibited sexual filamentation [32], [33]. To understand how blue light inhibits filamentation, we set up a genome wide mutagenesis screen and identified mutants suppressing the *CWC1* light-dependent overexpression phenotype [34]. One of the suppressors, AY18, restored mating filamentation and also showed dramatic de-repression of monokaryotic fruiting. Further characterization confirmed that a mutation of *C. neoformans SSN8* Mediator homologue is responsible for its phenotypes [34].

Sequence analysis revealed that *C. neoformans SSN8*, encoding a 439 amino-acid protein with two introns, is a putative Cyclin-like component of the RNA polymerase II holoenzyme. The conserved Cyclin box (46–122 amino acid) and CCL1 domain (244–280 amino acid) are identified. *C. neoformans* Ssn8 also contains a predicted PEST-rich region (298–315 amino acid) which has been known to be responsible for the degradation of Ssn8 in *S. cerevisiae* [23]. Homologues of the Ssn8 protein are widely present among diverse organisms. Overall identity is low among these proteins and high in the conserved domain regions. Unlike other ascomycetous homologues, no predicted destruction box (RxxL) is found in the *C. neoformans* Ssn8 protein [23], [25], [27]. The homologue most closely related to *C. neoformans* Ssn8 is the um06212 protein of *Ustilago maydis*; they shares 28% overall identity and 52% identity in the Cyclin box. *C. neoformans* Ssn8 protein also shares 18% and 19% overall identities respectively with *S. cerevisiae* Ssn8 and *Fusarium verticillioides* Fcc1, and 39% and 44% in the Cyclin box. Phylogenetic tree based on the whole protein sequence was generated (Fig. S1A) and amino acid sequence alignment of the Cyclin domains among the Ssn8 homologues is shown (Fig. S1B). *C. neoformans* Ssn8 also shows similar close relationship with the basidiomycetous homologues.

To confirm *C. neoformans SSN8* gene is a Mediator homolog, functional complementation was conducted in *S. cerevisiae*. *S. cerevisiae ssn8* mutant exhibits a flocculation phenotype when cultured in liquid medium. The *C. neoformans SSN8* gene was expressed under control of the *GAL1* promoter in pYES2 and transformed into the *S. cerevisiae ssn8* mutant and the transformants recovered the flocculation phenotype when grown in SD medium containing 2% galactose (Fig. S2). These results indicated that *C. neoformans SSN8* gene, encoding a functional mediator protein, is able to complement the phenotype of *S. cerevisiae ssn8* mutant.

### Deletion of *SSN8* Does Not Affect General Growth Capability, But Influences Galactose Utilization in *C. neoformans*

To determine the functions of *SSN8* in *C. neoformans*, *ssn8* null mutant and overexpression strains were generated. Southern blot analysis was carried out to confirm the strains (Fig. S3A and S3B). Real-time PCR analysis was also conducted to verify the levels of *SSN8* transcript. The results indicated that *SSN8* expression was not detected in the mutants and highly elevated *SSN8* transcript levels, at least 40 fold greater than the wild type, were detected in the overexpression strains (Fig. S3C).

**Figure 1:**
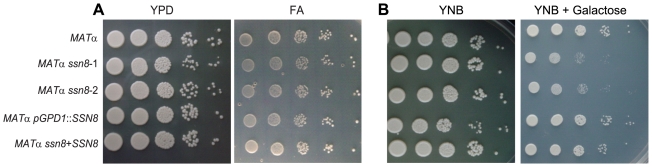
Deletion of *SSN8* does not affect general growth, but influences galactose utilization in *C. neoformans*. Strains subjected to cell growth assay were serially diluted and spotted onto YPD and FA (A), and YNB containing glucose and galactose (B) media. The cultures were incubated at 30°C in the dark for 2–5 days and photographed.

To further verify if deletion of *SSN8* affects the general growth capability of *C. neoformans*, we carried out cell growth assays on different media (Materials and Methods S1). When serially diluted yeast cells were spotted onto YPD rich and FA starvation media, the *ssn8* mutants and reconstituted strains all grew to the same extent as the wild-type strains (Fig. 1A). The *SSN8* overexpression strains also showed normal growth (Fig. 1A). We also examined the growth of mutants at 37°C and 39°C. No difference in growth rate was detected between the *ssn8* mutant and wild-type strain under these high temperature conditions (Fig. S3D). Therefore, deletion of the *SSN8* gene does not affect the general growth ability of *C. neoformans*.

Since *S. cerevisiae* Ssn8 is known to involve in the regulation of carbon utilization, we also determined whether *SSN8* deletion affects carbon utilization. The wild-type strains, *ssn8* mutants and *SSN8* overexpression strains were grown on YNB medium supplemented with different carbon sources, including glucose, sucrose, glycerol, ethanol, galactose, and sodium acetate. The *ssn8* mutants showed no growth defect on different carbon sources except for galactose-containing medium (Fig. 1B; data not shown). In addition, we also examined the expressions of *HXT* (a hexose transporter, CNB02680) and *SNF1* in the *C. neoformans MAT*α strains including three *ssn8* mutants in YPD liquid medium. Elevated transcript levels of these genes were detected in the mutants (Table S1). These results suggest that *C. neoformans* Ssn8 protein is possibly involved in the regulation of carbon, specifically galactose, utilization.

### Heterothallic a–α Mating Is Negatively Regulated by *SSN8*

In our previous study, *SSN8* was identified as a gene whose mutation restored mating filamentation of the *CWC1* light-dependent overexpression phenotype [34]. We postulated that *C. neoformans* Ssn8 may play a negative role in sexual differentiation. Hence, mating assays were conducted to determine the role of *SSN8* in the heterothallic **a**–α mating process. On V8 plate mating, both the *ssn8* unilateral and bilateral mutant crosses showed early formation of mating filaments at 8 h post incubation (Fig. S4A). However, upon further incubation filamentation around the edges of mutant mating spots was less pronounced when compared to the wild-type spot (Fig. S4B).

**Figure 2:**
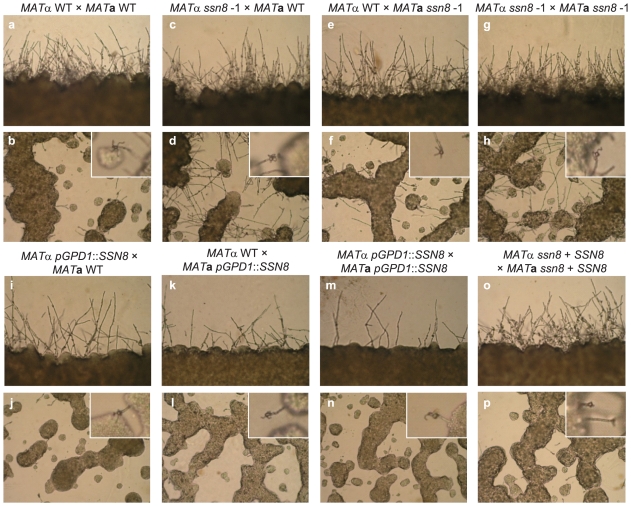
Mutation of the *SSN8* gene enhances heterothallic a-α mating responses. The *MAT*α and *MAT* **a** wild-type, *ssn8* mutant, and reconstituted strains were crossed as indicated. Mating was conducted on microscope slides coated with V8 agar at 26°C under light conditions and photos were taken from different areas of mating culture 44 h post incubation at 100× magnification under a microscope. Mating filaments around the edges of mating colonies were shown in upper panels (a, c, e, g, i, k, m, and o) and filamentation in the middle of mating culture was shown in lower panels (b, d, f, h, j, l, n, and p) at magnifications of 100× and 200× respectively. Insets showed chains of basidiospores and basidia at 400× magnification.

To more precisely examine the effect of *SSN8* deletion on mating, we further conducted a slide mating assay by diluting and spreading mating cells onto V8 agar film on microscope slides. In the unilateral (Fig. 2d and f) and bilateral mutant (Fig. 2h) crosses, more filaments were consistently observed in the diluted middle parts of mating cultures throughout the process when compared to the wild-type cross (Fig. 2b). More filaments at the edges of mutant mating spreads were also observed at early stages of the mating process. However, similar filamentation levels were seen in the mutant and wild-type crosses at later times (Fig. 2a, c, e, and g). Bilateral crosses between the *MAT*α and *MAT* **a** *SSN8* reconstituted strains (Fig. 2o and p) showed a filamentation pattern similar to the wild-type. In contrast, unilateral and bilateral crosses involving the *MAT*α and *MAT* **a** *SSN8* overexpression strains consistently exhibited less filamentation than those of the wild-type crosses when observed in the plate mating assay (Fig. 2i–n). Although filamentation levels were altered in crosses of mutant and overexpression strains, the timing for initiation of basidia formation in these crosses occurred around 36 h post incubation, which was similar to that in the wild-type cross. Fruiting structures produced by these crosses appeared normal (insets in the photos of Fig. 2). Based on our mating assays, we have demonstrated that deletion of the *SSN8* gene results in enhanced mating filamentation, whereas artificially elevating the *SSN8* transcript level reduces mating filamentation, suggesting that *C. neoformans* Ssn8 plays a negative role in heterothallic **a**–α mating process.

### Same-Sex Mating Is Dramatically De-repressed in the *MAT*a and *MAT*α *ssn8* Mutants

**Figure 3:**
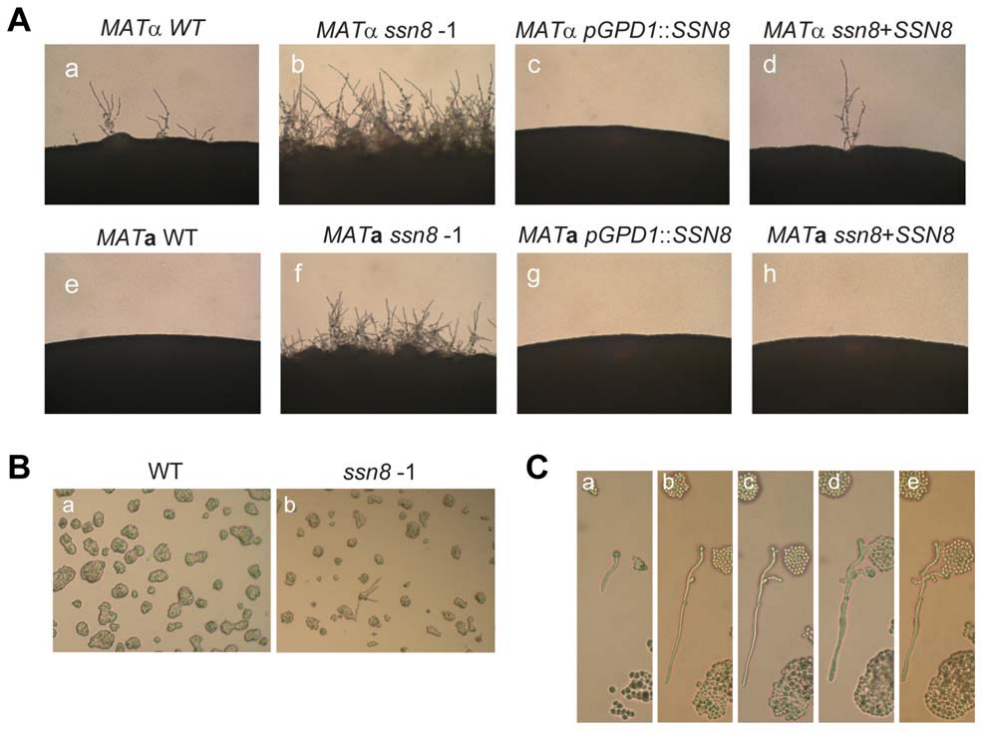
Same-sex mating is dramatically de-repressed in the *MAT*α and *MAT*a *ssn8* mutants. (A) The *MAT*α and *MAT* **a** wild-type, *ssn8* mutants, reconstituted strains, and *SSN8* overexpression strains were spotted on filament agar plate and incubated at 26°C under light conditions. Photos were taken after 5 days at 100× magnification. (B) The wild-type (a) and *ssn8* (b) cells were grown on microscope slides coated with FA agar film. Monokaryotic filaments were produced from the *ssn8* mutant cells. Photo was taken at 12 h post incubation at 100× magnifications. (C) Development of monokaryotic filament from a single enlarged *ssn8* mutant cell was monitored on microscope slide coated with FA agar at 12 h (a), 24 h (b), 36 h (c), 50 h (d) and 60 h (e) post incubation at 200× magnification.

Same-sex mating, also called monokaryotic fruiting, occurs in same mating type cells, predominantly in the *C. neoformans MAT*α strains [30]. A mutation of the *SSN8* gene by T-DNA integration, or by homologous replacement, in the *CWC1* overexpression strain surprisingly showed de-repression of same-sex mating [34]. We examined the effect of *SSN8* mutation on this process in the *MAT*α and *MAT* **a** wild-type strain background. Same-sex mating is a slow differentiation process in *C. neoformans*, and the serotype D *MAT*α wild-type strain JEC21 started to produce filaments on FA medium after 2 days and filamentation was clearly seen 5 days post incubation (Fig. 3Aa). In contrast, the *MAT* **a** wild-type strain JEC20 failed to undergo same-sex mating and no filament was observed after 5 days (Fig. 3Ae) and even after a prolonged incubation. As expected, same-sex mating was significantly de-repressed not only in the *MAT*α *ssn8* mutants but also in the *MAT* **a** *ssn8* mutants. Filamentation took place earlier in the *ssn8* mutant strains and more filaments were observed 2 days post incubation when compared to the wild-type strains. After 5 days, both *MAT*α and *MAT* **a** *ssn8* mutants showed profuse monokaryotic filaments, although more pronounced in the *MAT*α strain (Fig. 3Ab and Af). This de-repression phenotype was fully reversed to the wild-type levels both in the *MAT*α and *MAT* **a** reconstitution strains (Fig. 3Ad and Ah). On the other hand, same-sex mating process was completely blocked by *SSN8* overexpression. Both *MAT*α and *MAT* **a** *SSN8* overexpression strains failed to undergo this fruiting process after 5 days (Fig. 3Ac and Ag). No filament was developed even after 2 weeks incubation (data not shown).

We further conducted the same-sex mating assay, with diluted *C. neoformans* cells, on microscope slides coated with FA agar. The results showed that no filament was found in the wild-type strain at 12 h, and even up to 60 h post incubation, whereas, monokaryotic filaments were easily observed in the *ssn8* mutants (Fig. 3Bb). In these mutants, filamentous structures were observed as early as 8 h post incubation. Interestingly, most filaments arose from cells of larger size than regular yeast cells and no cell-cell conjugation event was found (Fig. 3C). These results suggest that *C. neoformans* Ssn8 negatively regulates the same-sex mating event in both *MAT*α and *MAT* **a** cells and plays a critical role in the control of this unusual sexual development.

### Mating Related Genes Are De-repressed in the *ssn8* Mutants

**Figure 4:**
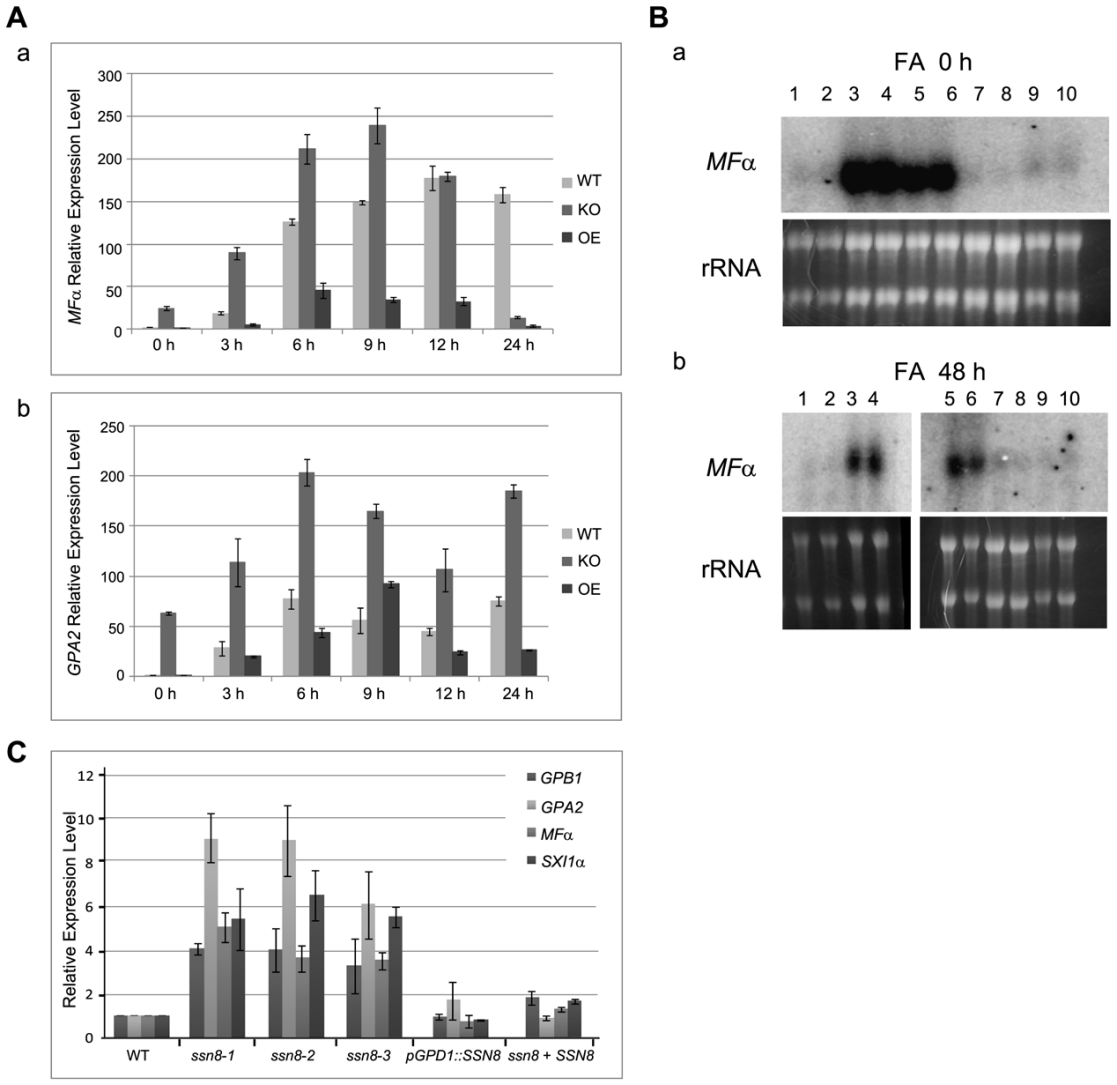
Mutation of the *SSN8* gene causes de-repression of genes involved in sexual development. (A) *C. neoformans* **a**-α bilateral crosses of the wild-type, *ssn8*-1 mutants and *SSN8* overexpressed strains were conducted on V8 agar medium in the dark and cells were collected at indicated time for RNA preparation. The abundance of the *MF*α (a) and *GPA2* (b) transcripts were measured by quantitative real time PCR analysis. (B) *C. neoformans* strains were grown on FA medium and cells were harvested at 0 (a) and 48 (b) h for RNA extraction. The expression of *MF*α was detected by northern blot analysis and RNA loading for each sample was shown by rRNA staining. Samples in lane 1 and 2 (*MAT*α wild-type), lane 3 and 4 (*MAT*α *ssn8*-1), lane 5 and 6 (*MAT*α *ssn8*-2), lane 7 and 8 (*SSN8* overexpression strain), lane 9 and 10 (*SSN8* reconstituted strain) are as indicated. (C) *C. neoformans MAT*α wild-type, *ssn8* mutants, reconstituted strain, and *SSN8* overexpression strain were grown in YPD liquid medium for 22 h. Cells were harvested and subjected to RNA extraction. Expression of the *GPB1*, *GPA2*, *MF*α and *Sxi1*α genes was measured by quantitative real time PCR analysis.

Observations of enhanced filamentation phenotypes of the *ssn8* mutants in the heterothallic **a**–α mating and also in the same-sex mating prompted us to further examine the expression of genes involved in these processes. Bilateral crosses of the wild-types, *ssn8* mutants, and *SSN8* overexpression strains were conducted on V8 agar media. Cells were collected at different time points and subjected to RNA extraction. The relative transcript levels of *MF*α and *GPA2* during **a**–α mating were measured by quantitative real time PCR analyses. *MF*α pheromone gene is known to be induced when strains of opposite mating type co-culture under nutritional starvation conditions [35]. *MF*α expression in the wild-type cross was low at 0 h and apparently induced to 18-fold at 3 h, and reached a peak of 178-fold at 12 h. Thereafter, the *MF*α transcript stayed high, but slightly declined to 158-fold at 24 h (Fig. 4Aa). In the *ssn8* bilateral mutant cross, the transcript level of *MF*α was 24-fold higher than that of the wild-type cross at 0 h. Mating induction of the pheromone gene in the mutant cross was more dramatic and its level was continually elevated and reached up to 238-fold at 9 h. At 12 h post incubation, the *MF*α level decreased in the *ssn8* mutant cross and showed similar expression level, 180-fold, to that in the wild-type cross. At 24 h, the *MF*α transcript level was significantly down-regulated and dropped to the basal level, 13-fold (Fig. 4Aa). In the bilateral cross of *SSN8* overexpression strains, the induction trend of *MF*α was generally similar over 24 hour period. However, the transcript level was significantly attenuated (Fig. 4Aa) when compared to the other two crosses.

*C. neoformans* G protein α subunit Gpa2 is a member of heterotrimeric G protein complex functioning upstream of the Cpk1 MAPK pheromone response pathway and its level is highly induced during mating process [36]. We also examined the *GPA2* transcript levels in the wild-type and *ssn8* mutant crosses and found that the overall *GPA2* expression patterns of these crosses were quite similar, and they both showed two peaks of *GPA2* transcript level at 6 and 24 h time points. However, higher induction levels were seen in the *ssn8* bilateral mutant cross throughout the 24 h period (Fig. 4Ab). In the bilateral cross involving the *SSN8* overexpression strains, the *GPA2* levels were lower than the wild-type cross at all time points except at its peak level at the 9 h time point (Fig. 4Ab).

Genes involved in heterothallic **a**–α mating also play roles in same-sex mating [30], [35]. Deletion of *SSN8* showed hyper-filamentation phenotype. To determine the *MF*α transcript level, *C. neoformans* strains were grown on FA agar and cells were harvested at 0 and 48 h for RNA analysis by Northern blot hybridization. *MF*α was barely detectable in the wild-type strain. In contrast, the *ssn8* mutants exhibited high levels of *MF*α pheromone expression at 0 and 48 h (Fig. 4Ba and Bb). As in the wild-type strain, *MF*α transcripts were also extremely low in the *SSN8* overexpression and reconstituted strains (Fig. 4Ba and Bb).

Our expression studies revealed that mating-related genes such as *MF*α and *GPA2* were highly elevated and still responsive during mating in the *ssn8* mutant cross. In addition, high pheromone transcript levels were also detected in the *ssn8* mutants during same-sex mating process. We speculated that Ssn8 may function as a general regulator repressing sexual-related genes during vegetative growth and therefore their expressions in YPD rich medium were further examined. *C. neoformans MAT*α strains including three *ssn8* mutants were grown in YPD liquid medium for 22 hours. RNA was extracted and subjected to real time PCR analyses. The transcript levels of *MF*α, *GPA2*, *GPB1*, *SXI1*α, *CPR2*, and other mating related genes among the *ssn8* mutants were significantly higher than those in the wild-type strain (Fig. 4C and Table S1). No significant differences were observed in the *SSN8* overexpression and reconstituted strains. In summary, our gene expression analyses have demonstrated that genes involved in sexual development are de-repressed in the *ssn8* mutants under non-mating and mating conditions. The *ssn8* mutants maintain the responsiveness to mating inducing signals, but the intensity and timing of gene expression pattern are changed.

### *C. neoformans* Ssn8 Plays Critical Roles Downstream of the Cpk1 MAPK Cascade and Ste12 in the Mating Signaling Pathway

**Figure 5:**
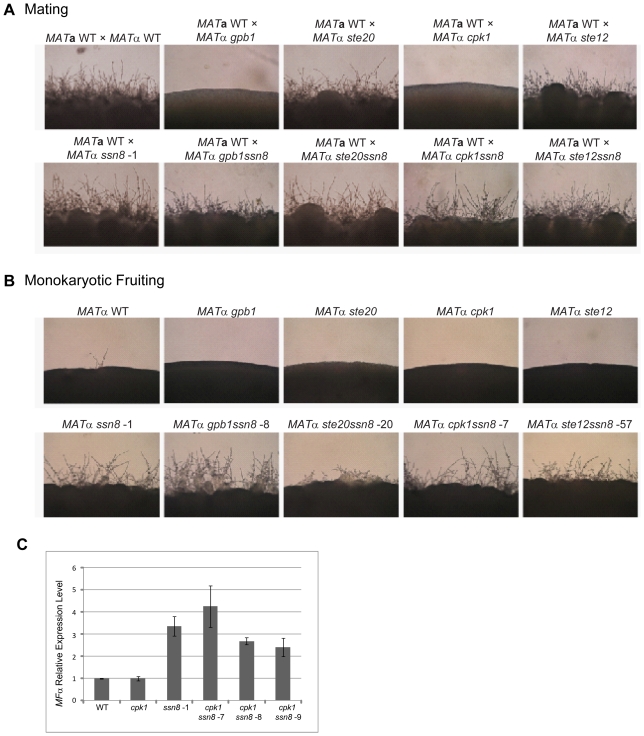
*C. neoformans* Ssn8 plays critical roles downstream of the Cpk1 MAPK cascade and Ste12 in the mating signaling pathway. (A) Unilateral crosses between the *MAT* **a** wild-type strain and *MAT*α wild-type or mutant strains as indicated were conducted on V8 medium at 26°C for 3 days in the light. (B) The *MAT*α wild-type, and *ssn8*, *gpb1*, *ste20*, *cpk1*, and *ste12*α single or double mutant strains were spotted on filament agar and incubated at 26°C for 3 days in the light. Photos were taken at 100× magnification. (C) The *MF*α pheromone gene was de-repressed in the *ssn8cpk1* mutants. The *MAT*α wild-type, *ssn8* and *cpk1* single mutants, and three independent *ssn8cpk1* double mutants were grown in YPD medium for 22 h. Relative expression levels of the *MF*α pheromone gene in these strains were determined by quantitative real time PCR analysis.

As demonstrated earlier, *C. neoformans SSN8* negatively regulates heterothallic **a**–α mating and same-sex mating processes and deletion of *SSN8* causes de-repression of sex-related genes (Fig. 2, 3 and 4). We therefore set out to determine the relationship between Ssn8 and components of the Cpk1 MAPK pheromone signaling pathway. We first conducted epistatic studies and generated double mutants of interest, including *gpb1ssn8*, *ste20ssn8*, *cpk1ssn8* and *ste12ssn8*. Their phenotypes were examined and compared with respective single mutants. As *gpb1* and *cpk1* mutants are sterile, the *gpb1ssn8* and *cpk1ssn8* double mutants produced profuse mating filaments, but slightly less than the *ssn8* mutant (Fig. 5A). The filamentation level in the *ste20ssn8* double mutant cross was comparable to that in the *ste20* mutant cross but slightly less than that in the *ssn8* mutant cross (Fig. 5A). In contrast, the *ste12ssn8* double mutant cross produced profuse filaments similar to the *ssn8* mutant but more than the *ste12* cross (Fig. 5A).

It is known that *GPB1* is required and *STE20*, *CPK1*, and *STE12* are all essential for the same-sex mating process [37]. Yet, we found that all the double mutants exhibited enhanced filamentation similar to the *ssn8* mutant, although to different extents (Fig. 5B). *STE12*, located in the mating type locus, is a major transcription regulator of same-sex mating. The *ste12ssn8* double mutant exhibited profuse monokaryotic filaments much the same as the *ssn8* mutant, which was in stark contrast to the sterile phenotype of the *ste12* mutant (Fig. 5B).

To support our phenotypic observation, we further examined the expression levels of *MF*α pheromone gene in the *cpk1* mutant background. The *cpk1* and *ssn8* single mutants, and three *cpk1ssn8* double mutants were grown in YPD liquid medium. RNA was extracted from these samples and subjected to quantitative real time PCR analysis. The results demonstrated that pheromone gene was de-repressed in the *ssn8* mutant, 3-fold higher than the wild-type strain; whereas, the *MF*α gene stayed at the basal level in the *cpk1* mutant. The expression levels of pheromone gene in three *cpk1ssn8* double mutants were all elevated and similar to that in the *ssn8* mutant (Fig. 5C). Taken together, our epistatic and gene expression studies suggest that Ssn8 plays critical roles downstream of the Cpk1 MAPK cascade and Ste12 and negatively regulates heterothallic **a**–α mating and same-sex mating processes.

### Disruption of *SSN8* Alters Cell Wall Structure and Integrity

**Figure 6:**
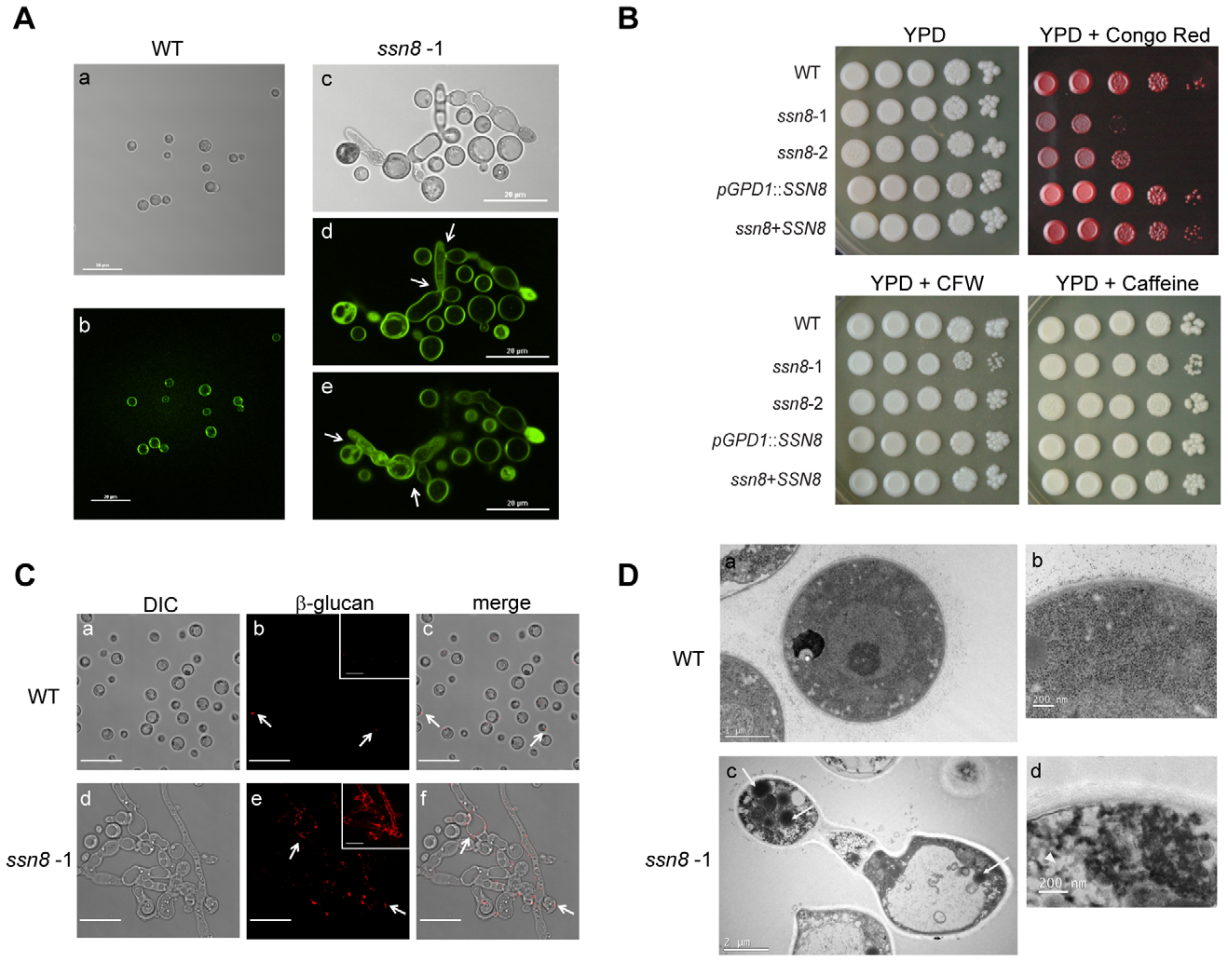
Cell wall structure and integrity are changed in the *ssn8* mutant strains. (A) The *C. neoformans* wild-type strain showed normal yeast cells (a, b), but the *ssn8* mutant displayed high percentage of morphological deformation (c, d, e) when grown in YPD liquid medium for 2 days. Fluorescence images of the same cells by Eosin Y staining are shown (b, d, e). Photos were taken at a magnification of 400× under a confocal microscope. (B) Equal number of the *MAT*α wild-type, *ssn8* mutants, *SSN8* overexpression and reconstituted cells were serially diluted and spotted on YPD medium and YPD supplemented with Congo red, calcofluor white (CFW) and caffeine. Plates were incubated at 30°C for 5 days under dark condition. (C) The wild-type and *ssn8* mutant strains were cultured in YPD liquid medium for 4 days. Cells were collected and stained with anti-β-1,3-glucan antibody and Cy3-labeled goat-anti-mouse secondary antibody for immunofluorescent observation. The DIC (a, d), fluorescent (b, e) and merged images (c, f) of the wild-type and *ssn8* mutant strains were shown. The projections of a series of z-axis fluorescent images were shown in the insets (b, e). Arrows indicate staining spots in the wild-type and *ssn8* mutant cells. Scale bar indicates 20 µm. (D) Transmission electron microscopy illustrates the wild-type (a, b) and the *ssn8* mutant (c, d) cells. Higher magnification images of the cells (b, d) were focused on the cell wall structure. Arrows indicate lipid droplets (c) and arrowhead indicates glycogen-like structure (d) in the *ssn8* mutant cells.

While growing the *ssn8* mutants in YPD liquid medium, we noticed some abnormally large aggregates of cells floating at the surface of the culture after 2 days of cultivation. Interestingly, upon microscopic observation, we found some of the *ssn8* mutant cells displayed an elongated and deformed morphology instead of the spherical yeast cells normally seen in the wild-type (Fig. 6Aa and Ac). Some of the elongated cells appeared to be septated but failed to separate. Under the same growth conditions, the reconstitution and overexpression strains both showed normal yeast cell morphology (data not shown). Quantification revealed that more than 17% of the *ssn8* mutant cells exhibited this atypical morphology (Table 1). To examine if cell wall structure was altered in *C. neoformans ssn8* mutants, we stained the cells with Eosin Y to visualize chitosan distribution in the cell wall [38]. In contrast to the uniform staining in the wild-type yeast cells (Fig. 6Ab), some of the *ssn8* mutant cells exhibited irregular patches by Eosin Y staining, suggesting the cell wall structure of the *ssn8* mutants was altered (Fig. 6Ad and Ae).

**Table 1:** Cell deformation rate of the *ssn8* mutants in the YPD liquid medium.

Strain	Deformation Rate	Strain	Deformation Rate
JEC20	0	JEC21	0.12%±0.24%
*MAT* **a** *ssn8*-1	43.53%±7.7%	*MAT*α *ssn8*-1	22.26%±4.42%
*MAT* **a** *ssn8*-2	26.53%±4.12%	*MAT*α *ssn8*-2	17.85%±3.44%
*MAT* **a** *pGPD1*::*SSN8*	0	*MAT*α *pGPD1*::*SSN8*	0
*MAT* **a** *ssn8*+ *SSN8*	0	*MAT*α *ssn8*+ *SSN8*	0

To further investigate the occurrence of structural modification or deformation, *C. neoformans* strains were grown in YNB and Filament liquid media. A high percentage of abnormal cells was found in the *ssn8* mutant cultures. The mutant cells grown in Filament liquid medium also showed hyper-elongated morphology (data not shown), whereas in YNB medium, the *ssn8* mutants exhibited round shape with a ragged cell surface (Fig. S5). When further stained with trypan blue and eosin Y, most of the ragged mutant cells showed strong staining with irregular patches, whereas the smooth and rounded wild-type cells showed uniform staining (Fig. S5).

Since mutation of *SSN8* caused abnormal morphology, we further examined if the *ssn8* mutants showed sensitivity to stress reagents. Yeast cells were serially diluted and spotted onto YPD medium containing different stress reagents, including Congo red, sodium dodecyl sulfate (SDS), caffeine, calcofluor white, and other osmotic, oxidative and nitrosative reagents. Their growths on these media were monitored. The results showed that the *ssn8* mutants were not sensitive to most of the stress reagents, only showing sensitivity to Congo red (Fig. 6B; Fig. S6). Congo red is known to interact with the important cell wall component β-1,3-glucan, and strains with cell wall defects will show sensitivity to this chemical [39].

Since *ssn8* mutants showed sensitivity to Congo red, we further examined the distribution of β-1,3-glucan by immunofluorescent staining. *C. neoformans* wild-type yeast cells showed relatively weak staining by anti-β-1,3-glucan antibody and only a small portion of cells showed fluorescent spots in the cell wall (Fig. 6Cb and Cc). In stark contrast, deformed yeast cells or filament structures of the *ssn8* mutant exhibited bright fluorescent staining in the cell wall (Fig. 6Ce and Cf). These observations confirm that the organization or distribution of β-1,3-glucan in the *ssn8* mutant cell wall is changed.

To reveal the detailed structural changes caused by the mutation of *SSN8*, we conducted transmission electron microscopy. In the *C. neoformans* wild-type strain, yeast cells overall displayed uniform cellular structures and compact cell wall organization (Fig. 6Da and Db). In contrast, the elongated and deformed *ssn8* mutant cells showed uneven cellular organization. Most mutant cells showed relatively light or uneven staining, and disordered structures or fractured organelles were found. Some mutant cells contained large vacuoles and round-shaped lipid droplets and glycogen (Fig. 6Dc and Dd), indicating that the mutant cells might be old or in different physiological stage due to increased storage components. Furthermore, the mutant cell wall appeared thicker and less condensed than the wild-type, consistent with the view that the cell wall structure and organization is changed in the *ssn8* mutant. Taken together, our findings indicate that a mutation of the *SSN8* gene affects cell wall structure and integrity in *C. neoformans*.

### The *ssn8* Mutants Display Invasive Growth on Rich Medium

**Figure 7:**
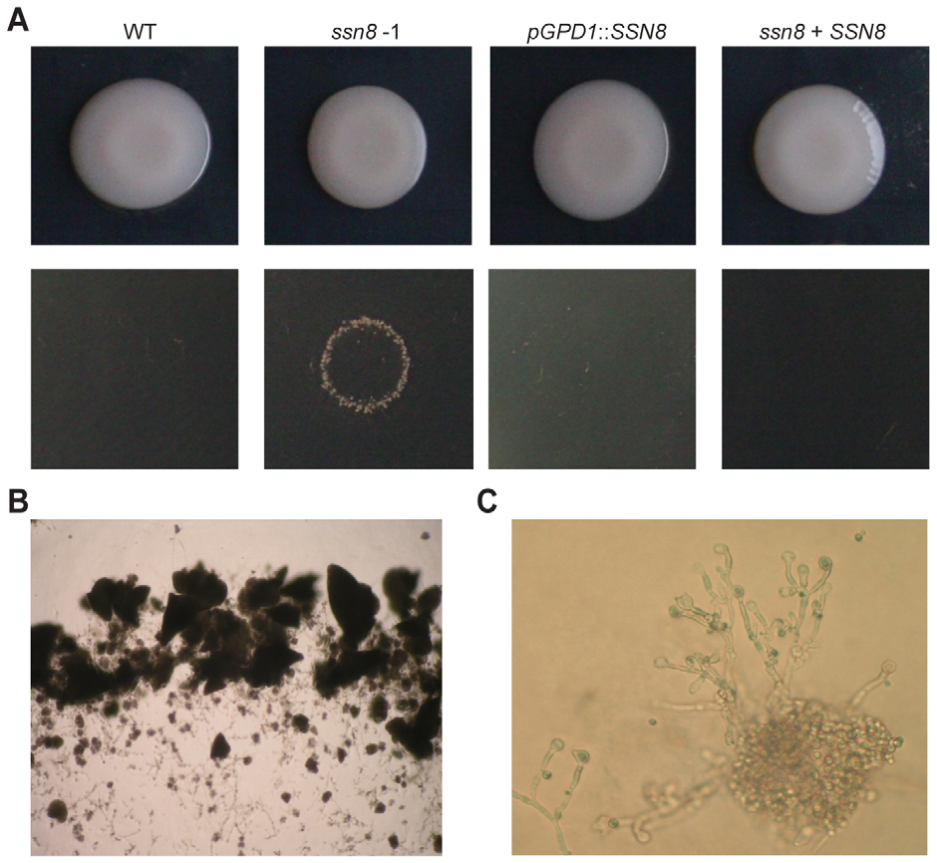
Deletion of *SSN8* displays invasive growth on YPD agar medium. (A) The *MAT* **a** wild-type, *ssn8* mutant, reconstituted, and *SSN8* overexpression strains were spotted on YPD agar medium and incubated at 30°C under light conditions for 10 days (top panel). After repeatedly washing the surface of medium, the *ssn8* mutant displayed invasive growth phenotype (bottom panel). (B) The cells remained on the agar surface were observed in the spot of the *ssn8* mutant after washing. Photo was taken at 40× magnification. (C) Filamentous deformed structures and cell aggregate are clearly seen in the *ssn8* mutant at 200× magnification.

*C. neoformans ssn8* mutants produced more abundant hyphae during sexual development processes. The *ssn8* mutants also showed elongated filament-like structures in liquid rich medium. We further examined if mutation of the *SSN8* gene caused any abnormal growth phenomenon on solid rich medium. To access the agar invasion phenotype, the wild-type, *ssn8* mutant, reconstituted, and overexpression strains were incubated on YPD medium (Materials and Methods S1). After 10 days of incubation, yeast colonies were repeatedly washed from the agar surface with sterile water. Interestingly, all the strains including the *SSN8* overexpression strains were readily removed from the agar surface except for the *ssn8* mutants (Fig. 7A). The remaining cells of the *ssn8* mutants were observed under a microscope and a mixture of hyphae and deformed yeast cells was found (Fig. 7B and C). We observed similar phenotypes when the *ssn8* mutants were grown on V8 or FA medium and hyphal invasion was clearly seen on these media (Fig. S7). Therefore, we conclude that Ssn8 also plays a negative role in regulating invasive hyphal growth in *C. neoformans*.

### Production of Melanin and Capsule Is Negatively Regulated by the *SSN8* Gene in *C. neoformans*

**Figure 8:**
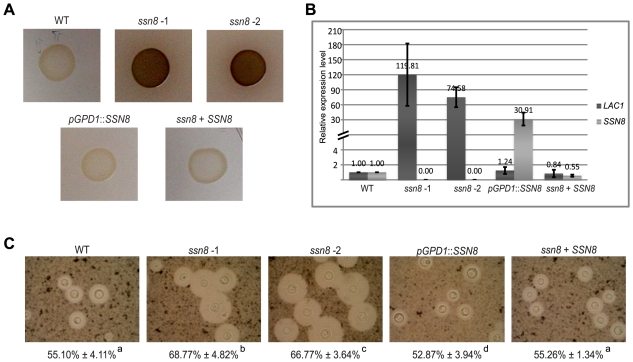
Formation of melanin and capsule is negatively regulated by the *SSN8* gene. (A) The *MAT*α wild-type, *ssn8* mutant, reconstituted, and *SSN8* overexpression strains were spotted on asparagine salt medium with L-DOPA and incubated at 26°C in the dark. Photos were taken after 6 days. (B) The expression levels of *LAC1* and *SSN8* in each strain were examined by quantitative real-time PCR analysis. All strains were grown in asparagine salt medium without glucose for 2 hours to induce *LAC1* expression. (C) The *MAT*α wild-type, *ssn8* mutant, reconstituted, and overexpression strains were grown in 0.1× Sabouraud medium buffered with 50 mM MOPS. Cultures were incubated at 30°C in the dark for 4 days. Cells were examined by negative staining with India ink under a microscope and photos were taken at 1000× magnification. The relative capsule sizes were determined by measuring 30 cells for each strain. The percentage represented the proportion of the capsule length relative to that of the whole cell. The same letters by the numbers indicated no significant difference at P = 0.05 according to Fisher's LSD method.

To determine if mutation of *SSN8* affects the virulence traits of *C. neoformans*, we examined *in vitro* production of melanin and capsule in the *ssn8* mutants. In the melanin production assay, *C. neoformans ssn8* mutants started to accumulate melanin earlier than the wild-type strain. By 6 days, melanin accumulation was apparent in the mutant strains, which was clearly different from the wild-type strain. No dramatic difference was observed among the wild-type, reconstitution, and *SSN8* overexpression strains (Fig. 8A). To confirm melanin accumulation in the *ssn8* mutants, we examined the expression levels of *LAC1* among these strains. We first checked the *SSN8* transcript level in the *ssn8* mutants and *SSN8* overexpression strains under induced conditions. No *SSN8* mRNA was detected in the *ssn8* mutants and high level of *SSN8* expression in the *SSN8* overexpression strain were confirmed (Fig. 8B). When laccase gene expression was examined, high levels of *LAC1* were detected in the *ssn8* mutants. Quantitative measurement by real-time PCR analysis revealed 120- and 75-fold higher levels than the wild-type in two independent *ssn8* mutants. The reconstitution and *SSN8* overexpression strains exhibited *LAC1* level similar to the wild-type strain (Fig. 8B).

To examine the formation of capsule in the *ssn8* mutants, strains were negatively stained by India ink after 4 days of induction and thirty cells from each strain were measured. The capsule sizes of the *ssn8* mutants were significantly larger than those of the other strains tested. The *SSN8* reconstitution and wild-type strains exhibited normal capsule sizes, whereas the overexpression strain displayed smaller capsule (Fig. 8C). These results indicated that *C. neoformans* Ssn8 also plays negative roles in the regulation of melanin and capsule production.

### *C. neoformans* Ssn8 Is Required for Virulence

**Figure 9:**
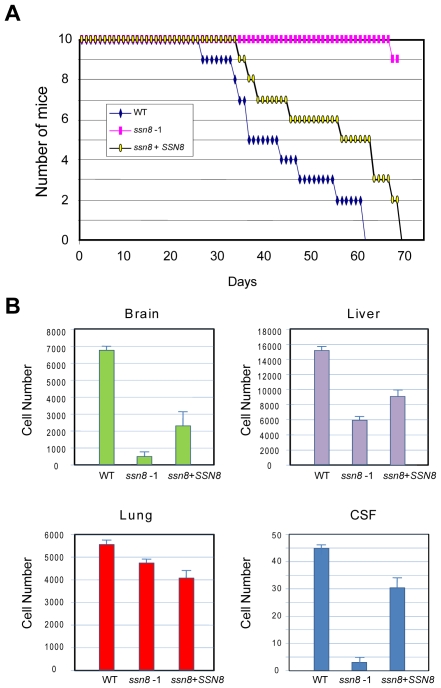
The *ssn8* mutant is attenuated for virulence. (A) Groups of ten C57LB/6 mice were infected with the *MAT*α wild-type, *ssn8* mutant, or the reconstituted strain and the survival of mice was monitored daily. The wild-type and reconstituted strains caused total morbidity in less than 70 days, whereas only one mouse was dead in the *ssn8* mutant group. (B) Groups of three C57LB/6 mice were infected with 10^6^ yeast cells of the *MAT*α wild-type, *ssn8* mutant, or the reconstituted strain via lateral tail vein injection for tissue burden studies. The tissues of brain, liver, lung and CSF were recovered after 24-hour infection. Quantitative fungal burden was performed by plating the tissue homogenates or CSF on YPD medium. The culture data from each sample was averaged and analyzed with a paired Student's t test. Data expressed as CFU were means of triplicate of the inoculated plates ± SD from two experiments (P<0.025). Notice that they are in different y-scales.

Mutation of *SSN8* resulted in increased accumulation of melanin and capsule, two well-known virulence factors of *C. neoformans*; however, the *ssn8* mutants also displayed altered phenotypes in other cellular and physiological processes. We next asked the question whether *C. neoformans* Ssn8 contributes to virulence by conducting animal survival, tissue burden, and cerebrospinal fluid analyses in a murine animal model. The *MAT*α wild-type, *ssn8* mutant, and reconstituted strains were each injected intravenously via the lateral tail vein into ten C57LB/6 mice each, and host health and survival were recorded daily. Initial death of mice infected with the wild-type or reconstituted strains were observed between 27 and 35 day, and subsequently all individuals of these two groups died within 70 days after inoculation (Fig. 9A). In contrast, mice infected with the *ssn8* mutant stayed healthy except for one death when close to day 70 since the inoculation (Fig. 9A).

To monitor the survival of *C. neoformans* strains inside the host body, we examined the yeast cell loads in different organs and cerebrospinal fluid after infection. Brain and lung were analyzed because they are the major targets of cryptococcal infection. In addition, cerebrospinal fluid was examined because it is the predisposed site of cryptococcosis in clinical presentations. We also analyzed liver because this organ is characterized by the presence of Kupffer cells; and Kupffer cells have been proven important for the clearance of circulating pathogens by extracellular or/and intracellular killing [40], [41]. On the day of the experiment, we injected 10^6^ yeast cells each of the wild-type, *ssn8* mutant, and reconstituted strains intravenously into the lateral tail vein of three C57LB/6 mice, individually. After 24 hours, the yeast cell loads in the brain, lung, liver and cerebrospinal fluid were examined. The CFUs of the wild-type strain showed highest infection in all four samples, whereas the *ssn8* mutant exhibited the lowest cell counts in the brain, liver and cerebrospinal fluid (Fig. 9B). The reconstituted strain partially complemented the mutant defect and restored infection and cell loads in the brain, liver and cerebrospinal fluid. Interestingly, no apparent difference in the cell numbers found in lung tissue was observed one day post-inoculation. However, there was a significant decrease in brain infection (Fig. 9B). These *in vivo* animal studies demonstrated that the *ssn8* mutant is defective in virulence and Ssn8 contributes to the virulence of *C. neoformans*.

### *C. neoformans* Ssn8 Does Not Directly Interact with the Cwc1 or Cwc2 Protein and Possibly Functions Downstream of the Cwc Complex

To identify components in the *C. neoformans* blue light mediated filamentation process, we conducted a genome wide mutagenesis and identified suppressor genes including *SSN8* in the screen [34]. These candidate genes may function either together with or downstream of the Cwc complex, or in an independent pathway. To determine whether Ssn8 physically interacts with Cwc1 or Cwc2, we conducted a yeast two-hybrid assay. Positive interactions were detected in the strains expressing AD-Cwc1 and BD-Cwc2, or AD-Cwc2 and BD-Cwc1 (Fig. S8B), as observed previously [32]. In contrast, no physical interaction was detected between Ssn8 and Cwc1/Cwc2 as no growth was observed in the strains containing AD/BD-Ssn8 and AD/BD-Cwc1/Cwc2 in combination (Fig. S8B). Control strains containing one *C. neoformans* gene and one empty vector also failed to grow. The yeast two hybrid analyses indicated that Ssn8 does not directly interact with Cwc1 or Cwc2.

**Figure 10:**
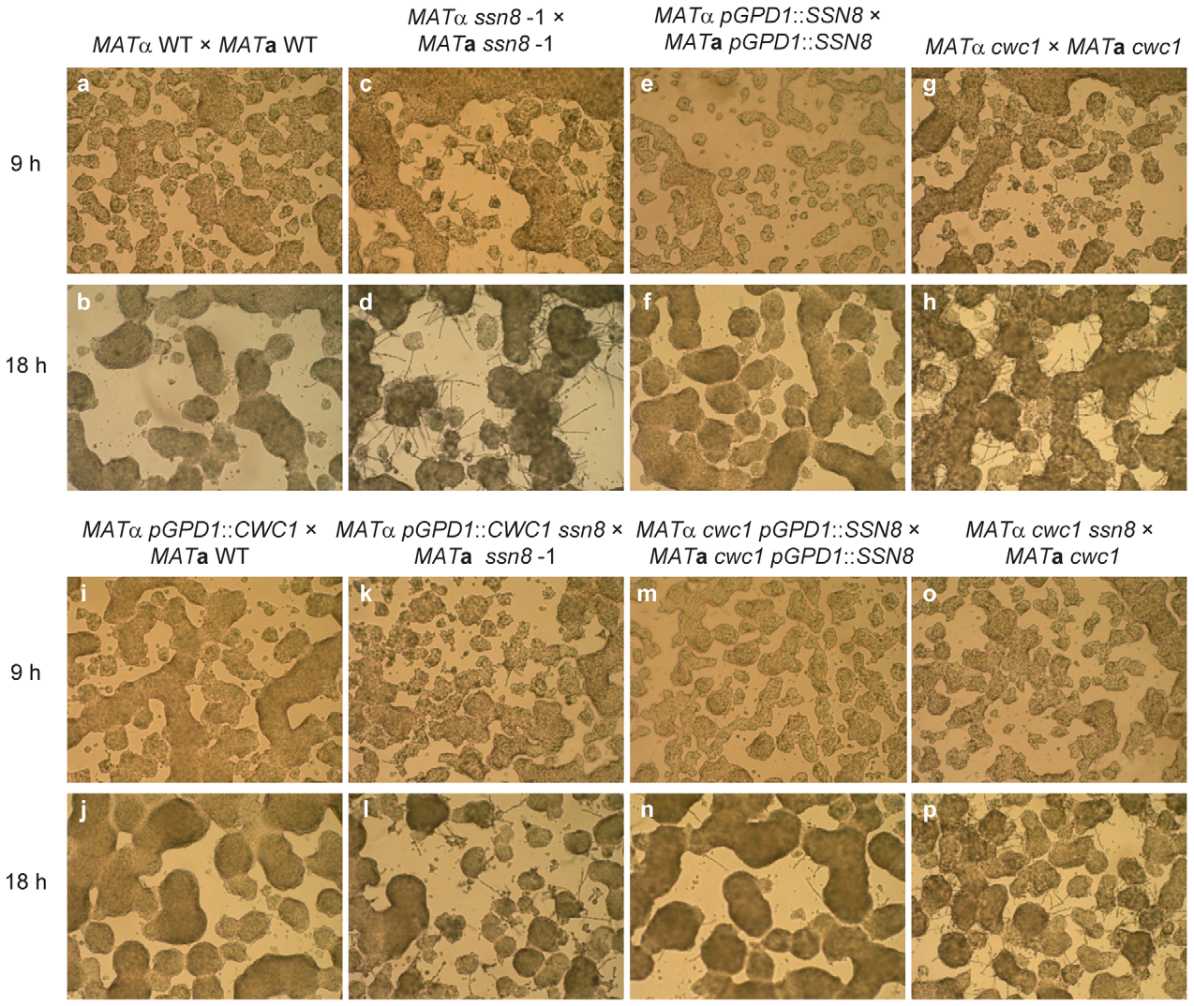
*C. neoformans* Ssn8 possibly functions at one of the major branches downstream of the Cwc complex. Epistasis analysis of the *CWC1* and *SSN8* genes was conducted by observing the mating phenotype on strains indicated on slide cultures under light condition. Photos were taken in the middle of the mating mixtures at 9 h (a, c, e, g, i, k, m, and o) and 18 h (b, d, f, h, j, l. n, and p) post incubation. Photos were taken at 100× magnification under a microscope.

We further determine the relationship between Ssn8 and Cwc1 by epistatic analysis. Strains containing *SSN8* and *CWC1* deletion, their respective overexpression, as well as their combined double mutants in the *MAT*α and *MAT* **a** background were tested in the slide mating assay. The timing and level of filamentation in the middle parts of diluted mating mixtures were recorded and compared. At 18 h post incubation, the bilateral mutant crosses of *ssn8* (Fig. 10d) and *cwc1* (Fig. 10h) showed more profuse filaments than the wild-type cross (Fig. 10b). At 9 h post incubation, however, filaments appeared in the *ssn8* unilateral or bilateral mutant cross (Fig. 10c; data not shown), while few or no filaments were produced in the wild-type (Fig. 10a) and *cwc1* mutant crosses (Fig. 10g). The bilateral cross of the *SSN8* overexpression strain showed few filaments (Fig. 10f), whereas no filaments (one filament occasionally observed in one field) were seen in the unilateral cross of the *CWC1* overexpression strain (Fig. 10j). Cross between the *MAT*α *CWC1* overexpression *ssn8* mutant and *MAT* **a** *ssn8* mutant (Fig. 10l) exhibited less filamentation than the *ssn8* bilateral mutant cross (Fig. 10d). These results confirmed that mutation of *SSN8* suppresses the light-dependent *CWC1* overexpression phenotype and Ssn8 does not function upstream of Cwc1.

Three additional pieces of evidence suggested that Ssn8 functions in one major branch downstream of the Cwc complex. First, the bilateral cross of the *cwc1 SSN8* overexpression strains (Fig. 10n) showed slightly more filaments than that of the *SSN8* overexpression bilateral cross (Fig. 10f). Second, filamentation level in the cross between the *MAT*α *cwc1 ssn8* mutant and *MAT* **a** *cwc1* mutant (Fig. 10p) was also slightly lower than that of the *cwc1* bilateral mutant cross (Fig. 10h). Third, the *ssn8* mutants were still responsive to light and their mating filaments were inhibited by light (data not shown). Based on the results from yeast two-hybrid and epistasis studies, we conclude that Ssn8 does not interact with the Cwc1 or Cwc2 protein and, instead, Ssn8 possibly functions in one major filamentation pathway downstream of the Cwc complex.

## Discussion

The Mediator complex is highly conserved among diverse organisms and is required for transcriptional regulation in eukaryotic cells [2], [4], [5], [42]. We studied light-regulated sexual development in *C. neoformans* and identified *SSN8* as a gene for a C-type cyclin Mediator whose mutation suppresses the *CWC1* light-dependent overexpression phenotypes [34]. In this study, we further characterized the roles of Ssn8 in details and found that this Mediator protein is important for diverse physiology in *C. neoformans*. Deletion of *SSN8* leads to pleiotropic phenotypes, including enhanced heterothallic **a**–α mating and same-sex mating responses, increased production of melanin and capsule, invasive growth, modified cell wall structure and integrity, and compromised virulence toward its mammalian host. Expression studies also reveal that genes involved in these processes are regulated by Ssn8. These results suggest that Ssn8 serves predominantly as a negative regulator in different physiological and differentiation processes in *C. neoformans*.

*C. neoformans* can undergo two types of sexual differentiation, heterothallic **a**–α mating and same-sex mating, and they have many features in common. They occur under similar conditions and share common signaling components including Cpk1 MAPK-mediated pheromone signaling pathway [30], [37]. It is also known that blue light inhibits both mating filamentation processes [32], [33]. Based on characterization of mutant phenotypes and the studies of gene expression in related strains under different conditions, we suggest that *SSN8* functions as a negative regulator in both processes. As for the mating phenotypes, the *ssn8* mutant strains exhibit enhanced mating responses including early onset and profuse production of dikaryotic filaments. In contrast, reduced mating filamentation is seen in the *SSN8* overexpression strains (Fig. 2). The *ssn8* mutant phenotypes correlate well with de-repression of sex-related genes under mating conditions (Fig. 4Aa and b). The absence of Ssn8 in the mating cells causes early de-repression of mating genes and consequently early appearance of filaments and better heterothallic **a**–α mating response are seen on V8 medium. Therefore, Ssn8 negatively regulates **a**–α opposite sex mating process. Furthermore, Ssn8 plays a dominant role in repressing same-sex mating process. The *MAT* **a** and *MAT*α *ssn8* mutants both show dramatic same-sex mating, whereas overexpression of *SSN8* blocks this process (Fig. 3A). Genes related to this same-sex mating process, especially the constitutively active pheromone receptor-like gene *CPR2*, are also de-repressed in the *ssn8* mutants (Table S2) [43]. Same-sex mating is a slow differentiation process which requires diploidization in fruiting cells of the same mating type [30]. This unusual mating event has been suggested as being responsible for disease outbreak in Canada [44]. In our study, detailed monitoring of the *ssn8* mutants on microscope slides revealed that monokaryotic filaments are mostly originated from cells with larger cell size (Fig. 3C). Characterization of the ploidy and other features of these large cells may reveal additional details of this differentiation process. Therefore, the *ssn8* mutants may serve as a platform to gain further insights into the nature of the same-sex mating process.

In YPD rich medium, genes involved in sexual development are normally repressed in the wild-type cells. Mutation of the *SSN8* gene releases repression, and sex-related genes including *GPB1*, *GPA2*, *MF*α and *SXI1*α are expressed (Fig. 4C; Table S1). However, successful mating between *ssn8* mutants of opposite mating types cannot occur under nutritional rich conditions (data not shown), suggesting additional signals or regulatory mechanisms are required for proper sexual development. In this study, we also examined the utilization of different carbon sources and found that *C. neoformans ssn8* mutants show growth defect on galactose medium, suggesting Ssn8 plays a positive role in galactose utilization. This is also in accord with the finding in *S. cerevisiae*, in which *SSN8* positively regulates the transcription of *GAL* genes [13], [14]. Furthermore, we found the transcript levels of two carbon utilization related genes were also elevated in the *ssn8* mutants under nutritionally rich condition (Table S1). Based on these results, we believe that *C. neoformans* Ssn8 is involved in the regulation of carbon utilization; nutritional repression of sexual development may at least partly be mediated by Ssn8 and its levels in mating cells may also play critical roles for proper sexual response.

*C. neoformans SSN8* gene was identified in our suppressor screen for possible components downstream of the Cwc complex in the blue light-inhibited sexual process [34]. We also determined the relationship between Ssn8 and Cwc complex as well as other known components in the pheromone signaling pathway by epistasis and gene expression studies (Fig. 5 and 10). We found that a mutation of *SSN8* in the *gpb1* and *cpk1* mutant background suppresses their sterile mating phenotypes and that the *ssn8ste12* double mutant also produces filaments similar to the wild-type strain, but more than the *ste12* mutant alone (Fig. 5A). Similar observations were also found in same-sex mating (Fig. 5B). Because Ssn8 has been shown to function as a global regulator, we cannot exclude the possibility that Ssn8 may regulate sexual development at multiple steps in the pheromone signaling pathway in *C. neoformans*. Our findings strongly suggest that Ssn8 plays critical roles downstream of the Cpk1 MAPK cascade and of the transcription factor Ste12. De-repression of sex-related genes in the *ssn8* mutants may be caused by first de-repressing a transcription factor downstream of Cpk1 or Ste12. Furthermore, direct physical interaction between Ssn8 and Cwc1/Cwc2 has not been detected by yeast two hybrid assay (Fig. S8A and S8B). Phenotypic comparison of strains with *CWC1/SSN8* related backgrounds indicated that Ssn8 also possibly regulates sexual development at one of major branches downstream of the Cwc complex (Fig. 10).

In *U. maydis*, Prf1 is a key transcription factor that binds specific pheromone response element in the promoter region of mating related genes. Prf1 is regulated by the MAPK and cAMP-PKA pathways both at the transcriptional and post-translational levels [45], [46]. A recent study has indicated that the *U. maydis* Ssn8 homologue functions downstream of Prf1, but is regulated independently by *kpp2/fuz7*, which are *C. neoformans CPK1/STE7* homologues [47]. In other words, *U. maydis* Ssn8 can be induced by Prf1, which is activated through an unknown kinase rather than by Kpp2 or other activation mechanisms. *C. neoformans* and *U. maydis* are evolutionarily related basidiomycetes. However, no role in sexual development has been revealed by the disruption of the *C. neoformans PRF1* homologue [48]. Instead of *PRF1*, a recent study has identified two transcription factors, Mat2 and Znf2, which function downstream of the Cpk1 MAPK cascade and regulate the pheromone response and hyphal development [48]. Furthermore, the expression of *CPR2* also depends on *MAT2* [43]. It will be of interest to determine the relationship between Ssn8 and these two regulators.

Invasive growth is thought to be a differentiation event for scavenging nutrients in response to nutritional starvation [49]. *C. neoformans ssn8* mutants show the invasive growth phenotype on rich medium (Fig. 7 and Fig. S7), suggesting that *SSN8* regulates the stress responses. In *S. cerevisiae*, nutrition deprivation leads to invasive growth in haploid cells or pseudohyphal growth in diploid cells, both of which allow cells to scavenge limited nutrients [50], [51]. In *S. cerevisiae*, *STE12*, a transcription factor downstream the *FUS3/KSS1* MAP kinase cascade, is required for invasive growth [52], [53]. *S. cerevisiae ras2* mutants also display reduced invasive growth [54]. Mutation of the *S. cerevisiae SSN8* or *SSN3* genes induces invasive growth in haploid strains [55]. Epistasis analysis suggests that *SSN8* and *SSN3* function downstream of *RAS2* to regulate invasive growth in *S. cerevisiae* [56]. Moreover, *S. cerevisiae* Ssn3 has been found to inhibit filamentous growth by phosphorylating Ste12 when cells are growing in rich medium [57], suggesting that nutritional signals may act through the C-type cyclin/CDK pair to regulate filamentous growth. In *C. neoformans*, the *ssn8* mutants display invasive growth on nutritional rich medium and have more severe invasion phenotypes in nutrition limited conditions (Fig. 7 and Fig. S7). The dominant active *RAS1* strain also exhibits invasive growth [58]. Deletion of *C. neoformans STE12* displays a defect in filamentous growth during the monokaryotic fruiting process and no role has been previously noted regarding invasive growth [59], [60]. Characterization of the *ssn8ste12* double mutant suggests that *SSN8* functions downstream of *STE12* in the mating processes. We are currently constructing the *ras1ssn8* double mutants and the *SSN8* overexpression mutant in the dominant active *RAS1* background and will use them to determine the relationship between Ssn8, Rasl and Ste12 in the invasive growth pathway of *C. neoformans*. These results also suggest that Ssn8 is an important switch which connects nutritional cues to developmental programs in *C. neoformans*.

*C. neoformans ssn8* mutants accumulate more melanin than the wild-type strain (Fig. 8A). Deletion of *SSN8* also results in dramatic de-repression of *C. neoformans LAC1* gene, the major gene responsible for melanin biosynthesis (Fig. 8B). Fungal Ssn8 homologues appear to be evolutionally conserved in regulating secondary metabolism including pigmentation and toxin production. *F. verticillioides FCC1* and *F. graminearum CID1* are both required for mycotoxin production, fumonisin and DON toxin respectively [25], [27]. Deletions of both genes also results in the accumulation of pigments and the gene expression in related processes is also altered. *F. verticillioides* Fck1, the Cyclin-dependent kinase partner of Fcc1 and *S. cerevisiae* Ssn3 homologue, has also been shown to coordinately regulate these processes with Fcc1 [26]. How Ssn8 is involved in the regulation of secondary metabolism, via a global or specific mechanism, requires further investigation.

In addition to melanin, the production of polysaccharide capsule is also negatively regulated by *SSN8*. The *C. neoformans ssn8* mutants show a larger capsule size than the wild-type strain (Fig. 8C). However, the *ssn8* mutant shows attenuated virulence in a murine model (Fig. 9A). Similar findings have been described in an *in vivo* screen of signature-tagged mutants [61]. In this study, several *C. neoformans* genes including *SSN8* are predicted to be required for virulence due to their reduced STM scores. The question of how Ssn8 contributes to the virulence is still an unresolved issue. One possible explanation for the virulence defect is that *ssn8* mutant cells may have difficulty in crossing the blood brain barrier due to their elongated cell morphology, or may be defective in putative brain predilection factor(s), which is yet to be demonstrated. Interestingly, it has been found that *C. neoformans* pseudohyphal structure can be induced by co-culturing with soil amoebae and the pseudohyphal strains are also attenuated in virulence [62]. Antigenic modifications in these cells have been suggested to evoke a stronger immune attack from the host [63]. Our studies have shown that *ssn8* mutants display abnormal cell morphology and sensitivity to Congo red medium (Fig. 6). The deformed *ssn8* mutant cells resemble pseudohyphal structures. This unusual cell morphology has also been described in the *C. neoformans rom2* deletion strain [64]. *ROM2* has been identified as a gene that is required for virulence in a nematode-based screen [65]. *ROM2*, the guanyl nucleotide exchange factor (GEF) of Rho1, is a regulatory component in the PKC-cell wall pathway. Rom2 can activate Pkc1 by turning Rho1 into the GTP-binding activated form that mediates the formation of cell wall structure. The *rom2* mutant displays normal virulence traits but has sensitivity to Congo red, and its avirulent phenotype has been confirmed in a murine model [65], [66]. These authors suggest that cell wall defect may be linked to the avirulence phenotype. Interestingly, Ssn8 has been shown to be regulated by Slt2 in the conserved PKC pathway in *S. cerevisiae* [22], [23]. Chitosan and β-1,3-glucan are both present in *C. neoformans* cell wall [38], [67]. Our studies using TEM and fluorescent microscopy have revealed that the cell wall organization and the distribution of chitosan and β-1,3-glucan are altered in the cell wall of *ssn8* mutant (Fig. 6). A study from another human fungal pathogen, *Candida albicans*, suggests that a *SSN8* mutation leads to increased exposure of β-D glucan, which is normally protected by an outer layer of mannoproteins. Such exposure elicits increased production of proinflammatory cytokines from primary macrophages that leads to enhanced immunity of the host and consequently, the *ssn8* mutant may become challenged and quickly lose the battle [68]. Our observations of immunofluorescent staining by anti-β-1,3-glucan antibody have revealed that β-1,3-glucan in the cell wall is also masked and not stained in the *C. neoformans* wild-type cells. In contrast, the *ssn8* mutant cells are heavily stained, suggesting that increased exposure of the β-1,3-glucan may also occur. Based on these results, we suggest that a mutation of *SSN8* causes modified cell wall structure and integrity that the mutant becomes increasingly immunity challenged and reduced fitness and consequently the *ssn8* mutant shows the attenuated virulence.

In summary, we have demonstrated that Ssn8 is an important negative regulator in both heterothallic **a**-α mating and same-sex mating processes. Our studies indicated that Ssn8 possibly functions at one of the major branches downstream of the Cwc complex and also plays critical roles downstream of the Cpk1-MAPK and Ste12 in the light-mediated sexual response pathway. Interestingly, Ssn8 acts as a global regulator in other physiological processes including virulence of this human pathogen. Our findings have also provided evidence that the member of the free module in the Mediator complex plays crucial roles in *C. neoformans*.

## Materials and Methods

### Strains and Growth Conditions

*Cryptococcus neoformans* congenic serotype D strains JEC20 (*MAT* **a**) and JEC21 (*MAT*α) were used as the wild-type strains [69]. The auxotrophic derivative strains such as JEC34 (*MAT* **a** *ura*5), JEC43 (*MAT*α *ura*5), and others were also used in this study [70]. All the strains utilized and generated in this study are listed in Table 2. *C. neoformans* strains were routinely cultured on YPD (1% yeast extract, 2% peptone and 2% dextrose) medium at 30°C. Synthetic media (SD) lacking specific nutrient supplement were utilized for genotype verification and selection of transformants [71]. V8 agar medium was used for heterothallic **a**–α mating assay [72]. Filament agar (FA, pH 5.0) was used for same-sex mating assay [73].

**Table 2:** Fungal strains used in this study.

Strain	Description	Reference
*Cryptococcus neoformans*		
JEC20	*MAT* **a** WT	[69]
JEC21	*MAT*α WT	[69]
JEC34	*MAT* **a** *ura*5	[70]
JEC43	*MAT*α *ura*5	[70]
WSC129	*MAT*α *gpb1::URA5 ura5*	[35]
RDC23	*MAT*α *ste12*α*::URA5 ura5*	[37]
RDC5	*MAT*α *cpk1::ADE2 ade2*	[37]
CSB7	*MAT*α *ste20*α*::URA5 ura5*	[82]
YKC7	*MAT*α *cwc1::URA5 ura5*	[33]
YKC29	*MAT* **a** *cwc1::URA5 ura5*	[33]
YKC38	*MAT*α *pGPD1::CWC1*+*URA5 ura5*	[33]
YSC1	*MAT*α *ssn8::NAT + pGPD1::CWC1-URA5 ura5*	[34]
YSC2-1	*MAT*α *ssn8::NAT* (*MAT*α *ssn8*-1)	This study
LIC1	*MAT*α *ssn8::NAT* #157(*MAT*α *ssn8*-2)	This study
YSC2-2	*MAT*α *ssn8::NAT* (*MAT*α *ssn8*-3)	This study
LIC2	*MAT*α *ssn8::NAT* #9	This study
YSC3-1	*MAT* **a** *ssn8::NAT* (*MAT* **a** *ssn8*-1)	This study
LIC3	*MAT* **a** *ssn8::NAT* #99 (*MAT* **a** *ssn8*-2)	This study
YSC3-2	*MAT* **a** *ssn8::NAT*	This study
LIC4	*MAT* **a** *ssn8::NAT#19*	This study
YSC9	*MAT*α *pGPD1::SSN8*-*URA5 ura5*	This study
YSC10	*MAT* **a** *pGPD1::SSN8*-*URA5 ura5*	This study
YSC11	*MAT*α *ssn8::NAT *+ *SSN8::HYG*	This study
YSC12	*MAT* **a** *ssn8::NAT *+ *SSN8::HYG*	This study
YSC7-1	*MAT*α *cwc1::URA5 ura5 ssn8::NAT*	This study
LIC5	*MAT*α *pGPD1::CWC1-URA5 ura5 ssn8::NAT*	This study
LIC6	*MAT* **a** *pGPD1::CWC1-URA5 ura5 ssn8::NAT*	This study
LIC7	*MAT*α *gpb1::URA5 ura5 ssn8::NAT* #7	This study
LIC8	*MAT*α *gpb1::URA5 ura5 ssn8::NAT #8*	This study
LIC9	*MAT*α *ste20*α*::URA5 ura5 ssn8::NAT* #10	This study
LIC10	*MAT*α *ste20*α*::URA5 ura5 ssn8::NAT* #20	This study
LIC11	*MAT*α *ste12*α*::URA5 ura5 ssn8::NAT* #57	This study
LIC12	*MAT*α *ste12*α*::URA5 ura5 ssn8::NAT* #21	This study
LIC13	*MAT*α *cpk1::ADE2 ade2 ssn8::NAT* #7	This study
LIC14	*MAT*α *cpk1::ADE2 ade2 ssn8::NAT* #8	This study
LIC15	*MAT*α *cpk1::ADE2 ade2 ssn8::NAT* #9	This study
LIC16	*MAT*α *pGPD1::CWC1*-*URA5 ura5 ssn8*Δ + *SSN8::HYG*	This study
LIC17	*MAT*α *pGPD1::CWC1*-*URA5 ura5 ssn8::NAT *+ *SSN8::HYG*	This study
*Saccharomyces cerevisiae*		
PJ69-4A	*MAT* **a** *trp1-901 leu2-3, 112 ura3-52 his3-200 gal4*Δ*gal80*Δ *LYS2::GAL1-HIS3 GAL2-ADE2 met2::GAL7-lacZ*	[81]
BY4742	*MAT*α *his3-1*, *leu2-0*, *ura3-0*, *lys2*	EUROSCARF, Franfurt, Germany
YNL025C	*MAT*α *his3-1*, *leu2-0*, *ura3-0*, *lys2*, *YNL025c::KanMX4*	EUROSCARF, Frankfurt, Germany

### Sequence Alignment of the Ssn8 Homologues and Generation of the Phylogenetic Tree

The Ssn8 homologues were identified using the BLASTP program against the NCBI GenBank database. The amino acids sequences were downloaded and subjected to sequence analyses. Sequence alignments and comparisons were conducted by ClustalX program [74]. Phylogenetic tree based on the maximum likelihood method was constructed by submitting to the on-line program provided by the Tree-Puzzle website (http://mobyle.pasteur.fr/).

### Complementation of the *S. cerevisiae ssn8* mutant

The cDNA of *C. neoformans SSN8* gene was subcloned into pYES2 yeast expression vector to generate pYES2::*CnSSN8*, which *SSN8* was expressed under control of the *GAL1* promoter. The pYES2::*CnSSN8* and pYES2 constructs were delivered into YNL025C by biolistic transformation. Transformants were selected on SD medium lacking uracil and screened by PCR. To examine the complementation phenotype, yeast cells were incubated in liquid SD medium lacking uracil but in the presence of glucose or galactose at 30°C overnight. Yeast cultures were then shaken and photographed after 90 min.

### Disruption and Reintroduction of the *C. neoformans SSN8* Gene

To disrupt the *C. neoformans SSN8* gene, an *ssn8::NAT* mutant allele previously constructed was used for transformation [34]. The disruption construct was delivered into the wild-type and other strains by biolistic transformation [75]. Transformants were selected on YPD medium with 100 µg/ml nourseothricin and verified by PCR and Southern blot analysis.

To generate the *SSN8* reconstitution construct, a 5810 bp genomic fragment containing the *SSN8* open reading frame and flanking regions was amplified by primers WC530 and WC469. The PCR product was purified, digested by XbaI and XhoI, and cloned into pJAF15 [76], which contains the hygromycin selectable marker. The *SSN8* reconstitution plasmid was biolistically transformed into the *ssn8* mutant strains in both *MAT* **a** and *MAT*α mating types. Transformants were selected on YPD with 200 µg/ml hygromycin, re-confirmed the nourseothricin resistance and finally verified by PCR and Southern blot analysis. Primers used in this study are listed (Table S2).

### Overexpression of the *C. neoformans SSN8* Gene

To generate the *C. neoformans SSN8* overexpression construct, a 2.0 kb fragment containing the *SSN8* open reading frame and 3′-flanking terminator sequences was amplified by primers WC445 and WC446. The fragment was digested with BamHI and SmaI, and subcloned into the pYKL8, which contains the *C. neoformans GPD1* promoter and *URA5* selectable marker [33]. The plasmid was sequence-verified and transformed into JEC34 (*MAT* **a** *ura*5), JEC43 (*MAT*α *ura*5), and other strains by biolistic transformation. Uracil prototrophic transformants were picked from SD plate lacking uracil, screened by PCR amplification, and finally verified by Northern blot and real-time PCR analyses.

### Northern Blot and Real-Time PCR Analyses

Total RNA was extracted from the *C. neoformans* samples using TRIzol total RNA isolation reagent (Invitrogen). Protocols for Northern blot hybridization were conducted as previously described [77]. Northeren hybridization probe for the pheromone genes was amplified by PCR primers WC572 and WC573.

Real-time PCR analysis was conducted according to the manufacturer's instructions (Applied Biosystems). Relative gene expression level was normalized with the constitutively expressed *C. neoformans GPD1* gene. Primers used for real-time PCR analyses are listed (Table S2).

### Examination for Cell Morphology

To examine cell morphology, *C. neoformans* strains were cultured in 50 ml YPD liquid medium at 30°C for 2 days. Ten microliters of cell suspension was taken out, spotted onto haemacytometer, and examined under a microscope (Olympus BX41). Deformed cells were counted and the deformation rate was expressed as the number of deformed cells divided by the total cell number.

To observe the distribution of chitosan in the cell wall, a described staining method was followed [38]. Briefly, the tested strains were grown in different liquid media at 30°C for 2 days, and 0.5 ml of cell suspension was centrifuged, washed once with sterile water, and resuspended in 0.5 ml McIlvaine's buffer (pH 6.0). Thirty microliters of eosin Y solution (5 mg/ml, Sigma) was added into cell suspension, gently shaken at 30°C for 30 min, and 0.5 ml trypan blue solution (0.4%, Sigma) was then added and incubated for another 10 min. Finally, the stained cells were washed and resuspended in 0.3 ml McIlvaine's buffer. Trypan blue and eosin Y staining were respectively examined by brightfield and fluorescence illumination under a confocal microscope (A1R, Nikon).

### Heterothallic a–α mating and Same-Sex Mating Assays

Two types of mating assays, plate and slide mating, were conducted. Strains subjected to mating assays were first streaked on YPD agar and grown at 30°C for 2 days. Single yeast colonies were picked and resuspended in sterile water. Strains of opposite mating type were mixed in equal amount for the heterothallic **a**-α mating assay, otherwise, cell suspensions of single mating type strain were directly conducted for the same-sex mating assay. Cells were cultured at 26°C in the light and dark. For plate mating assay, cell suspension was spotted onto V8 agar/FA plate. For slide mating assay, thin film of V8 agar/FA was made on microscope slides. Cell suspension was added at one side of the slide and the slide was then tilted to make the mixture spread across the V8 agar/FA film. Mating filamentation and fruiting structures were observed and photographed under a microscope (Olympus BX41).

### Melanin Production Assay

Strains subjected to melanin assay were first cultured in 5 ml YPD liquid medium and grown at 30°C for overnight. Cells were harvested by centrifugation, washed twice with sterile water, and finally resuspended in sterile water. Five microliters of cell suspension from each tested strain was spotted on asparagine salt agar with L-DOPA (100 mg/l) and kept in the dark at 26°C [78]. Melanization of the colony was checked after 2–5 days and recorded by a digital camera (Sony DSC-S85). Strains subjected to the study of *LAC1* gene expression were initially grown overnight in 5 ml YPD liquid medium. Cells were collected, washed twice with sterile water, and transferred to asparagine broth medium (0.1% glucose). Cultures were further incubated at 30°C until mid-log phase. Cells were then harvested, repeatedly washed, transferred to asparagine broth medium without glucose, and grown for additional 2 hours to induce *LAC1* gene expression [79].

### Phenotypic and Quantitative Analyses of Capsule Formation

To examine capsule formation, fresh single yeast colony of tested strains from YPD plate was inoculated in 5 ml 0.1× Sabouraud medium buffered with 50 mM MOPS for capsule induction [80]. Cultures were grown at 30°C for 3–4 days and the formation of capsule was examined by negative staining with India ink under a microscope (Olympus BX41). To conduct the quantitative analysis of capsule formation, same growth procedure was followed. The thickness of capsule layer around individual cell was manually measured. The relative ratio of capsule size was expressed as the difference in diameter between the whole cell and cell body without capsule divided by the diameter of the whole cell. Thirty randomly selected cells were measured for each tested strain and statistical analysis was performed by Fisher's LSD method.

### Sample Preparations for Gene Expression Analyses

Strains subjected to gene expression analyses were first grown on YPD agar for 2 days. For YPD culture, single colony was inoculated in 5 ml YPD liquid medium and incubated overnight at 30°C. Then 1.5 ml culture was inoculated into 13.5 ml YPD liquid medium and grown at 30°C for additional 22 hours. Cells were harvested and immediately frozen in liquid nitrogen for RNA extraction. For same-sex mating samples from filament agar, single colony was inoculated in 5 ml YPD liquid medium and incubated overnight at 30°C. The whole culture was transferred into 45 ml YPD liquid medium and grown with agitation at 30°C for an additional 22 hours. Cells were harvested, washed once with sterile water, and resuspended in 12.5 ml sterile water. Cell density was counted and adjusted to 10^8^ cells/ml. Then 20 µl of cell suspension was spotted onto FA medium and a total of 10 spots were added on each plate. Plates were incubated at 26°C in the darks for up to 2 days. Cells were collected from the surface and immediately frozen for RNA extraction. For heterothallic **a**–α mating samples from V8 agar plate, YPD culture was similarly grown as the same-sex mating samples. Cells of opposite mating type strain were harvested, washed once with sterile water, counted and mixed in 1 to 1 ratio, and adjusted to a final density of 1.2×10^8^ cells/ml. A portion of cell suspension was centrifuged, frozen and used as time 0 control sample for RNA extraction. Then 20 µl from the cell mixture was spotted onto V8 agar medium and a total of 10 spots were added on each plate. Plates were incubated at 26°C in the darks for up to 24 hours. Cells were collected from the surfaces of the plates at 3, 6, 9, 12 and 24 hours post incubation and immediately frozen in liquid nitrogen for RNA extraction.

### Yeast Two Hybrid Assay

Yeast two hybrid assay was followed as described [81]. The cDNAs of *SSN8*, *CWC1*, and *CWC2* were amplified and cloned into pGAD-C1 and pGBDU-C1 respectively. All the inserted sequences were confirmed by sequencing. The respective prey and bait constructs with a different insert as well as the empty vectors were co-transformed into the yeast strain, PJ69-4A, to detect the interaction. Transformants were selected on SD medium lacking uracil and leucine to confirm the successful transformation of the two constructs. Yeast two hybrid assay was conducted on SD medium lacking uracil, leucine and histidine medium but supplemented with 50 mM 3-Amino-1,2,4-triazole (3-AT) to detect positive interaction.

### Generation of the Double Mutant Strains for Epistasis Analysis

The *ssn8* disruption construct with nourseothricin selection marker was biolistically transformed into the *cwc1* and *gpb1* mutant strains, as well as into the *CWC1* overexpression strain. Transformants were selected on YPD medium with 100 µg/ml nourseothricin and verified by PCR. Other double mutants, including *ste12ssn8*, *ste20ssn8*, *cpk1ssn8*, and *SSN8* overexpression in the *cwc1* mutant background, were generated by crossing the strains with appropriate genotypes. Progeny with expected genotypes were screened by PCR and verified by growing on selective media.

### Immunofluorescent Study

The strains subjected to immunofluorescent observation were cultured in YPD liquid medium for 4 days at 30°C. Five hundred microliters of cells were collected and washed twice with PBS/ 0.1% BSA, and followed by staining with 20 µg/ml of anti-β-1,3-glucan antibody (Biosupplies Inc., Parkville, Australia) at 25°C for 3 hours. Cells were then briefly washed with PBS/0.1% BSA three times and further incubated with 10 µg/ml Cy3-labeled goat-anti-mouse secondary antibody at 25°C for 2 hours. After washing with PBS/0.1% BSA, stained samples were observed under DeltaVision Core deconvolution microscope (Applied Precision).

### Transmission Electron Microscopy

The *C. neoformans* strains subjected to TEM examination were first cultured in YPD liquid medium for overnight. Cells were collected by centrifugation at 10,000 g for 30 min and fixed overnight in 2.5% glutaraldehyde at 4°C. Cells were then washed with 0.1 M phosphate buffered saline (PBS) three times, and further subjected to postfixation in 1% osmium tetroxide for 80 min. After being washed in 0.1 M PBS three times, cell samples were dehydrated in a graded series of ethanol and finally washed in 100% ethanol three times. Cells were embedded in resin, and 70 nm thin sections were prepared. Samples were examined and photographed in JOEL JEM-1400 electron microscope.

### Animal Virulence Assays

Mouse survival measurements were performed with 6-week old C57LB/6 mice. Three groups of 10 mice were infected with a total of 10^6^ yeast cells of the *MAT*α wild-type, *ssn8* mutant, and reconstituted strain via lateral tail vein injection, individually. Survival was monitored daily, and moribund mice or those in pain were sacrificed by CO_2_ inhalation. The survival of groups of 10 infected mice was determined for the infected mice.

For the analyses of tissue burden and cell load in the cerebrospinal fluid, three groups of three 6-week old C57LB/6 mice were infected with a total of 10^6^ yeast cells of the *MAT*α wild-type, *ssn8* mutant, and reconstituted strain via lateral tail vein injection. After 24 hours infection, mice were euthanized which was conducted by anesthetizing i.p. by ketamine and xylazine (100 and 10 µg per body weight, respectively) and exsanguinating by cardiac puncture. The cerebrospinal fluid was drawn, and the brain, lung and liver tissues were harvested. Organ tissues were resuspended in PBS and homogenized. Quantitative cultures were performed by plating dilutions of the tissue homogenates and cerebrospinal fluid on YPD plates.

## Supporting Information

Figure S1 **Phylogenetic analysis and sequence alignment of the** ***C. neoformans*** **Ssn8 and related homologues.** (A) Phylogenetic tree was constructed by the maximum likelihood method based on the protein sequences of the Ssn8 homologues. The names of the organisms were abbreviated and the gene name or locus number for each homologue was indicated. Af, *Aspergillus fumigatus*; An, *Aspergillus nidulans*; Ca, *Candida albicans*; Cc, *Coprinopsis cinerea*; Ce, *Caenorhabditis elegans*; Cn, *Cryptococcus neoformans*; Dm, *Drosophila melanogaster*; Gm, *Gibberella moniliformis*; Hs, *Homo sapiens*; Kl, *Kluyveromyces lactis*; Lb, *Lodderomyces elongisporus*; Le, *Laccaria bicolor*; Mg, *Magnaporthe grisea*; Mglo, *Malassezia globosa*; Nc, *Neurospora crassa*; Sc, *Saccharomyces cerevisiae*; Um, *Ustilago maydis*; Xl, *Xenopus laevis*. Numbers above each branch are bootstrap values based on 1000 replications. (B) Amino acid sequence alignment of the cyclin domain among the Ssn8 homologues.
(TIF)

Figure S2 **The** ***C. neoformans SSN8*** **gene complements the flocculation phenotype of the** ***S. cerevisiae ssn8*** **mutant.** Yeast strains were grown in SD medium containing 2% glucose (Lane 1–2) and SD medium lacking uracil containing 2% glucose (Lanes 4 and 6) or 2% galactose (Lane 3 and 5) at 30°C overnight. The cultures were mixed and photographed after 90 min. Yeast strains: 1, wild-type (BY4742); 2, *ssn8* mutant (YNL025C); 3, 4, *ssn8* mutant+pYES2; 5, 6, *ssn8* mutant+pYES2::*CnSSN8*.
(TIF)

Figure S3 **Deletion of the** ***C. neoformans SSN8*** **gene in the wild-type strains, and** ***C. neoformans ssn8*** **mutant strains display no growth defect under high temperature conditions.** (A) An *ssn8::NAT* disruption allele was generated by replacing the *SSN8* coding region with the *NAT* selectable marker [34]. (B) Southern hybridization was conducted to verify the *ssn8* mutants and reconstituted strains. Genomic DNA from the *MAT*α wild-type (Lane 1), *ssn8* mutants (Lane 2–4), and reconstituted strain (Lane 5) and the *MAT* **a** wild-type (Lane 6), *ssn8* mutants (Lane 7–9), *ssn8* mutant with ectopic integration (Lane 10), and reconstituted strain (Lane 11) was digested with HindIII and hybridized with the probe as indicated in (A). (C) The relative expression levels of *SSN8* in the *MAT*α strains as indicated were detected by quantitative real-time PCR and normalized with the *C. neoformans GPD1* gene. (D) The 10-fold diluted cells were spotted on YPD medium. Plates were kept at high temperature as indicated.
(TIF)

Figure S4 **Early formation of dikaryotic filaments is observed in the bilateral** ***ssn8*** **mutant cross.** (A) The *ssn8* bilateral mutant cross displayed early formation of mating filaments compared to the wild-type cross. Mating assay was conducted on V8 plate under light condition. Photos were taken at different time points. (B) The mating reactions of strains as indicated were conducted on V8 plates and photos were taken at 18 h post incubation under light condition.
(TIF)

Figure S5 **Deletion of** ***SSN8*** **results in modified cell wall structure.** The *ssn8* mutant cells exhibited ragged surface (d) compared to smooth surface of the wild-type cells (a) in YNB medium. High proportion of the *ssn8* mutant cells (e) were heavily stained by trypan blue, but only few in the wild-type cells (b). Uniform staining by eosin Y was found in the wild-type cells (c); whereas, irregular patches of eosin Y staining was observed in the *ssn8* mutant cells (f). All photos were taken at 400× magnification under a confocal microscope.
(TIF)

Figure S6 ***C. neoformans ssn8*** **mutant strains exhibit no growth defect under different stress conditions.** The 10-fold diluted cells were spotted on YPD medium or YPD media containing different stress reagents, including 3 mM NaNO_2_ (A), 1 M KCl, 0.03% SDS, and 3 mM H_2_O_2_ (B).
(TIF)

Figure S7 **Deletion of** ***SSN8*** **induces invasive growth on different media.** (A) The *ssn8* mutant strains displayed severe invasive growth on V8 medium at 26°C under light condition after 24 h. Colonies were washed off by sterile water and photos were taken. (B) *C. neoformans* strains as indicated were incubated on filament agar at 26°C for 9 days. The margins of the *ssn8* mutant colonies showed more abundant hyphae than those of the wild-type, reconstituted and overexpression strains. (C) The 9-day fruiting colonies on filament agar were washed by sterile water and photos were taken. The *ssn8* mutants showed more severe invasive growth than the wild-type strains.
(TIF)

Figure S8 ***C. neoformans*** **Ssn8 does not directly interact with the Cwc1 or Cwc2 protein.** The coding sequences of *SSN8*, *CWC1* and *CWC2* were cloned into pGAD-C1 or pGBDU-C1 and cotransformed into *S. cerevisiae* strain PJ69-4A to test physical interaction. Transformants were streaked on SD-ura-leu medium (A) and SD-ura-leu-his+50 mM 3-AT medium (B) and positive interaction was detected only between Cwc1 and Cwc2.
(TIF)

Materials and Methods S1 (DOC)

Table S1 **The relative expression levels of** ***C. neoformans*** **genes in the wild-type and** ***SSN8*** **-related strains in YPD liquid medium.**
(DOC)

Table S2 **Primers used in this study.**
(DOC)
