# Supplementary figures and images for: Cryptococcus neoformans Mediator Protein Ssn8 Negatively Regulates Diverse Physiological Processes and Is Required for Virulence
Source: PLoS One. 2011 Apr 29;6(4):e19162. doi: 10.1371/journal.pone.0019162 (PMC3084776; doi:10.1371/journal.pone.0019162)

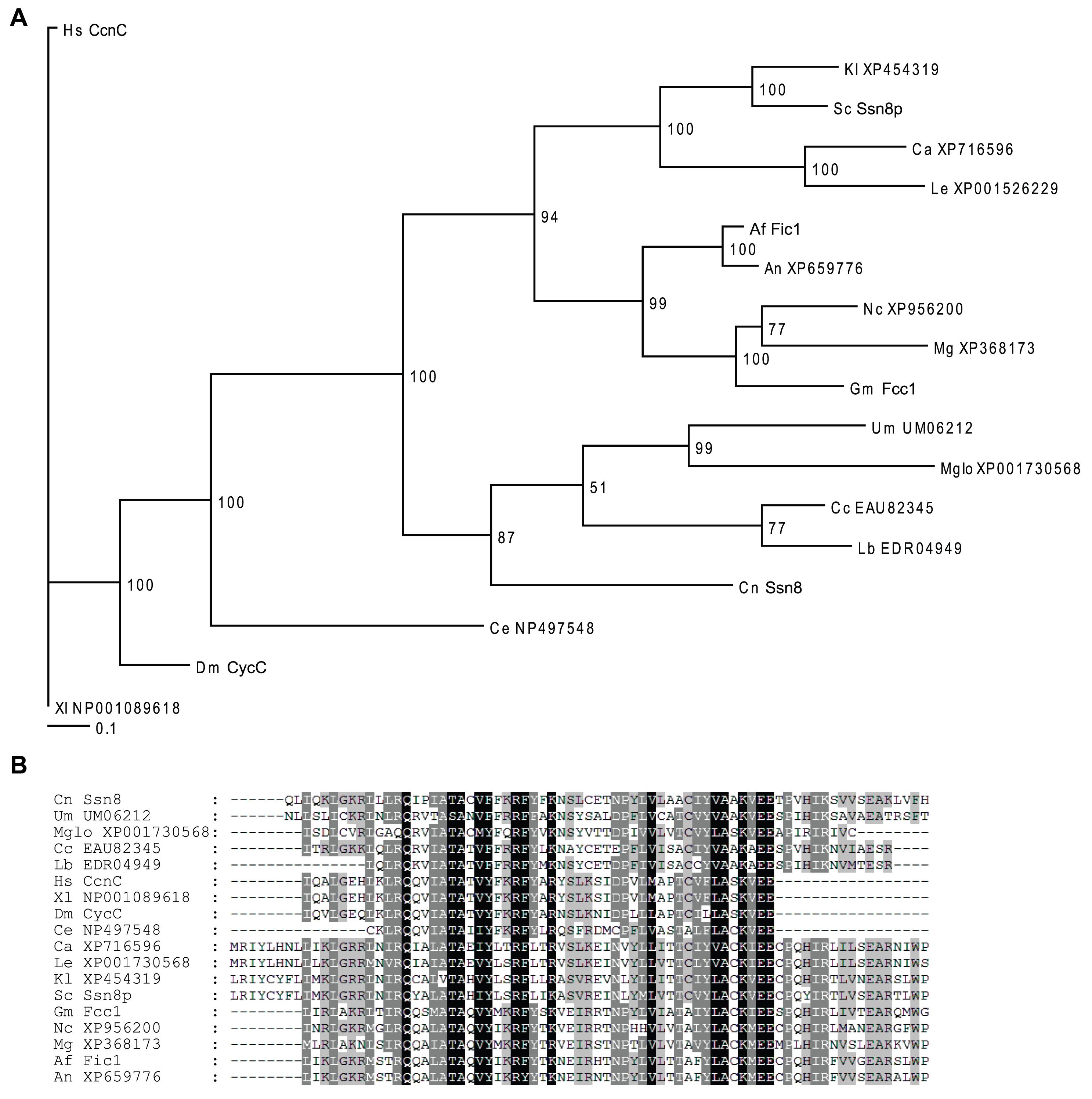

Supplement: Figure S1 — Phylogenetic analysis and sequence alignment of the C. neoformans Ssn8 and related homologues. (A) Phylogenetic tree was constructed by the maximum likelihood method based on the protein sequences of the Ssn8 homologues. The names of the organisms were abbreviated and the gene name or locus number for each homologue was indicated. Af, Aspergillus fumigatus; An, Aspergillus nidulans; Ca, Candida albicans; Cc, Coprinopsis cinerea; Ce, Caenorhabditis elegans; Cn, Cryptococcus neoformans; Dm, Drosophila melanogaster; Gm, Gibberella moniliformis; Hs, Homo sapiens; Kl, Kluyveromyces lactis; Lb, Lodderomyces elongisporus; Le, Laccaria bicolor; Mg, Magnaporthe grisea; Mglo, Malassezia globosa; Nc, Neurospora crassa; Sc, Saccharomyces cerevisiae; Um, Ustilago maydis; Xl, Xenopus laevis. Numbers above each branch are bootstrap values based on 1000 replications. (B) Amino acid sequence alignment of the cyclin domain among the Ssn8 homologues. (TIF) [file pone.0019162.s001.tif]

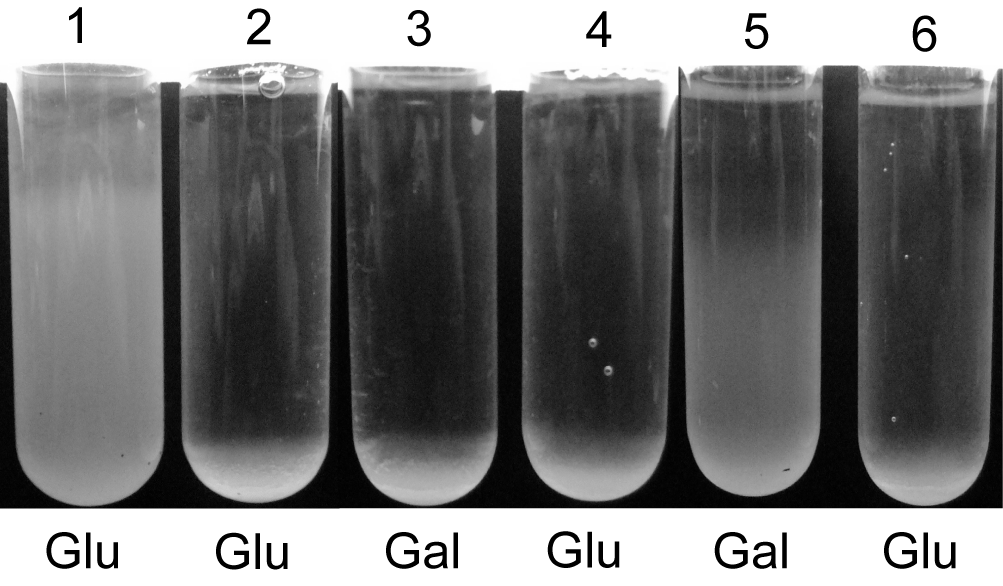

Supplement: Figure S2 — The C. neoformans SSN8 gene complements the flocculation phenotype of the S. cerevisiae ssn8 mutant. Yeast strains were grown in SD medium containing 2% glucose (Lane 1–2) and SD medium lacking uracil containing 2% glucose (Lanes 4 and 6) or 2% galactose (Lane 3 and 5) at 30°C overnight. The cultures were mixed and photographed after 90 min. Yeast strains: 1, wild-type (BY4742); 2, ssn8 mutant (YNL025C); 3, 4, ssn8 mutant+pYES2; 5, 6, ssn8 mutant+pYES2::CnSSN8. (TIF) [file pone.0019162.s002.tif]

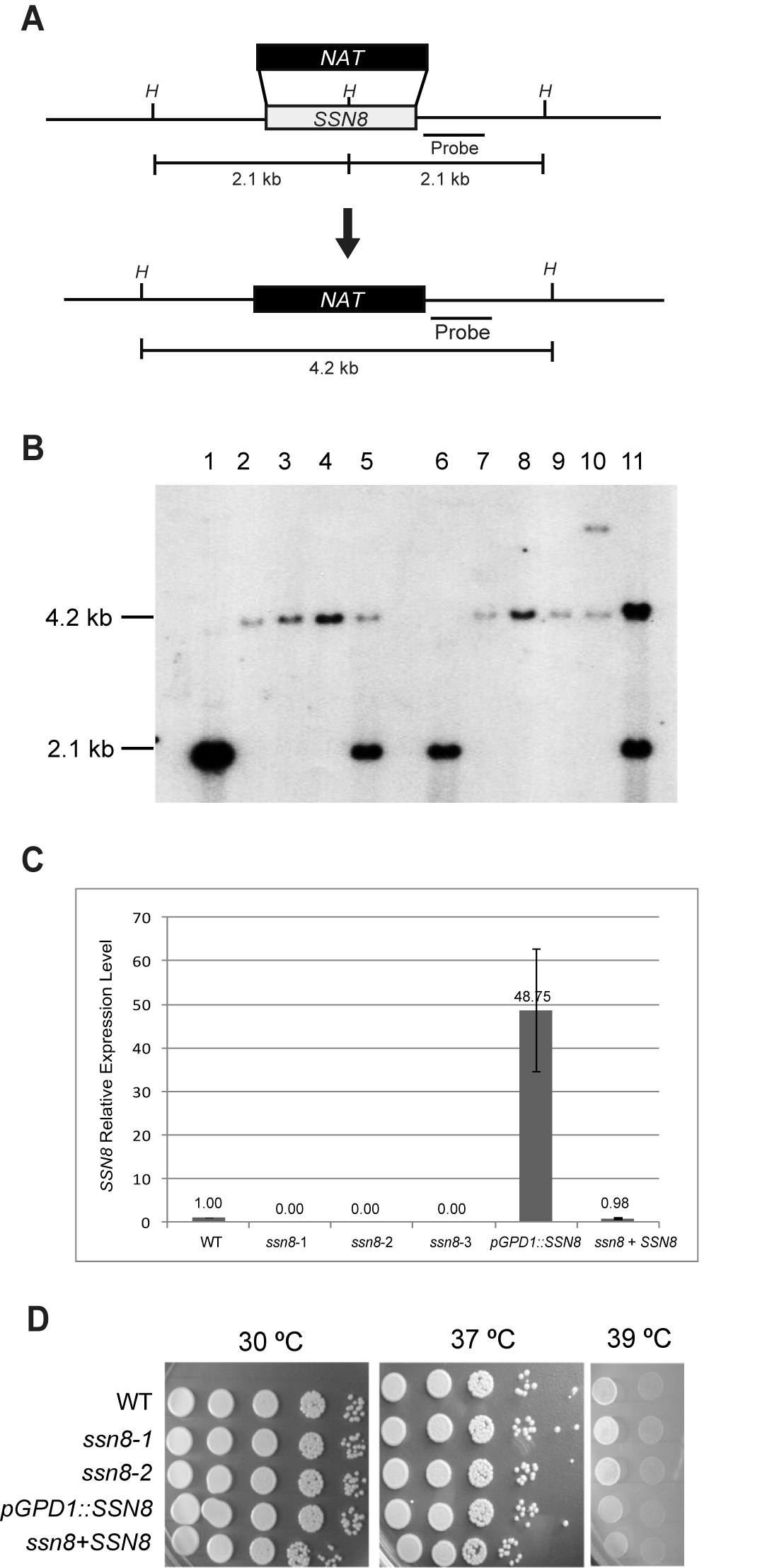

Supplement: Figure S3 — Deletion of the C. neoformans SSN8 gene in the wild-type strains, and C. neoformans ssn8 mutant strains display no growth defect under high temperature conditions. (A) An ssn8::NAT disruption allele was generated by replacing the SSN8 coding region with the NAT selectable marker [34]. (B) Southern hybridization was conducted to verify the ssn8 mutants and reconstituted strains. Genomic DNA from the MATα wild-type (Lane 1), ssn8 mutants (Lane 2–4), and reconstituted strain (Lane 5) and the MAT a wild-type (Lane 6), ssn8 mutants (Lane 7–9), ssn8 mutant with ectopic integration (Lane 10), and reconstituted strain (Lane 11) was digested with HindIII and hybridized with the probe as indicated in (A). (C) The relative expression levels of SSN8 in the MATα strains as indicated were detected by quantitative real-time PCR and normalized with the C. neoformans GPD1 gene. (D) The 10-fold diluted cells were spotted on YPD medium. Plates were kept at high temperature as indicated. (TIF) [file pone.0019162.s003.tif]

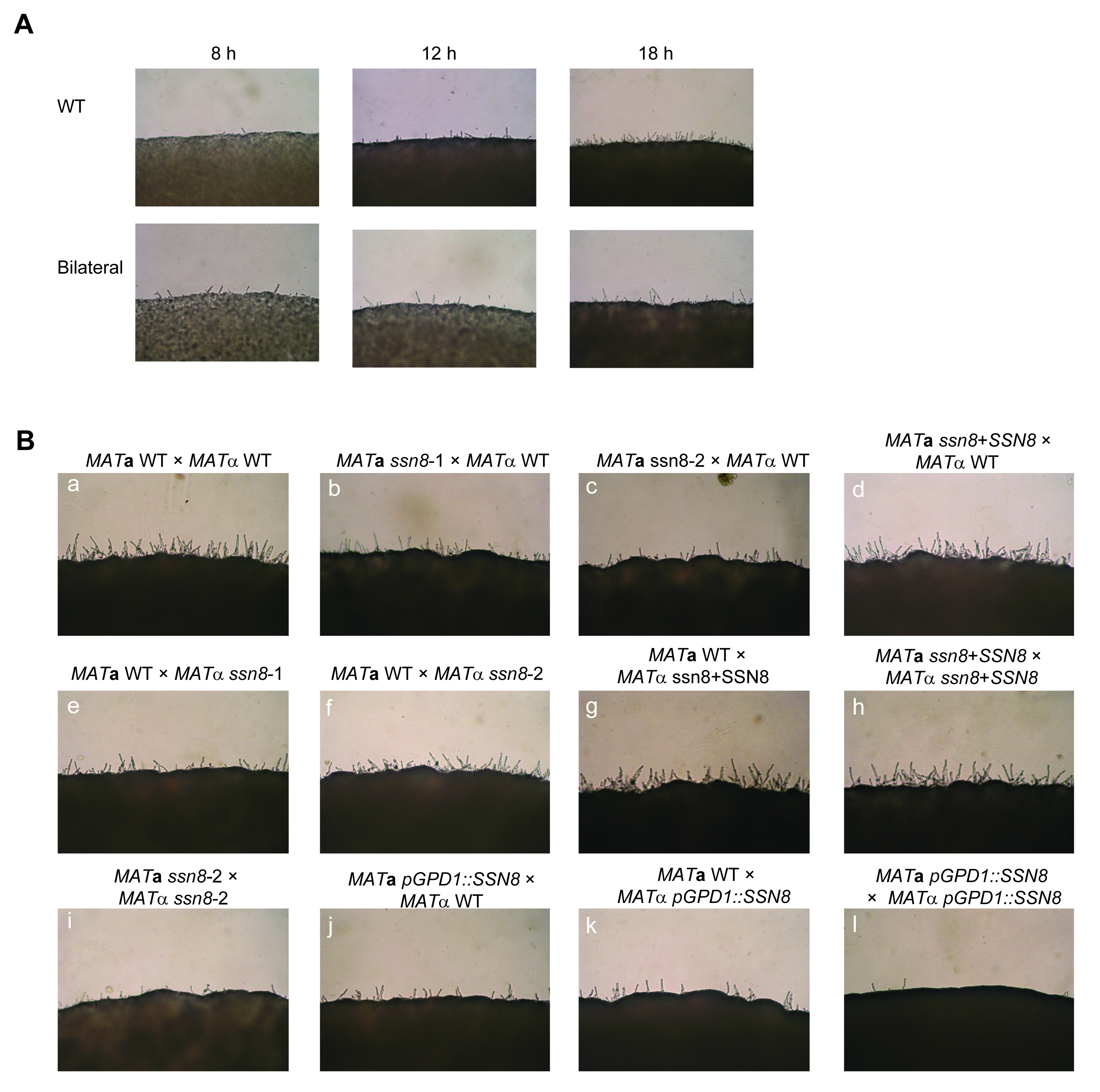

Supplement: Figure S4 — Early formation of dikaryotic filaments is observed in the bilateral ssn8 mutant cross. (A) The ssn8 bilateral mutant cross displayed early formation of mating filaments compared to the wild-type cross. Mating assay was conducted on V8 plate under light condition. Photos were taken at different time points. (B) The mating reactions of strains as indicated were conducted on V8 plates and photos were taken at 18 h post incubation under light condition. (TIF) [file pone.0019162.s004.tif]

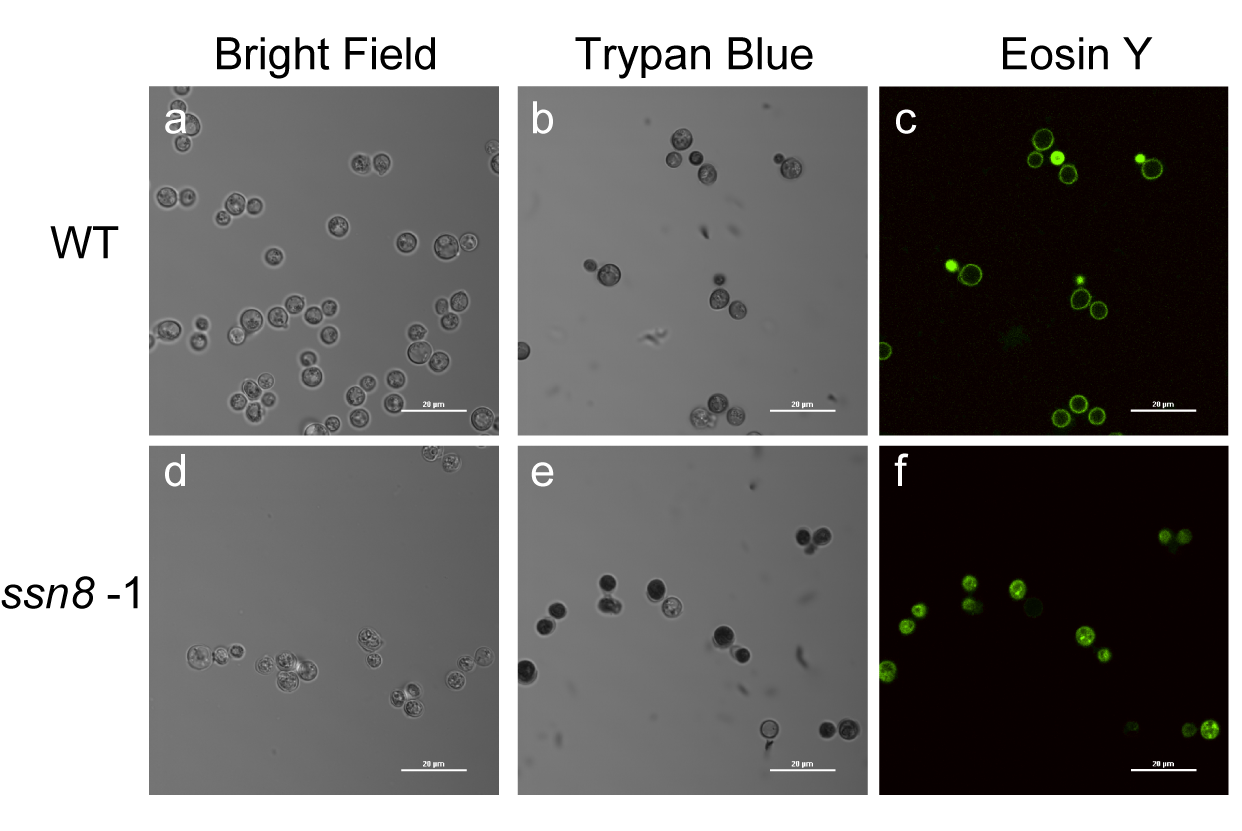

Supplement: Figure S5 — Deletion of SSN8 results in modified cell wall structure. The ssn8 mutant cells exhibited ragged surface (d) compared to smooth surface of the wild-type cells (a) in YNB medium. High proportion of the ssn8 mutant cells (e) were heavily stained by trypan blue, but only few in the wild-type cells (b). Uniform staining by eosin Y was found in the wild-type cells (c); whereas, irregular patches of eosin Y staining was observed in the ssn8 mutant cells (f). All photos were taken at 400× magnification under a confocal microscope. (TIF) [file pone.0019162.s005.tif]

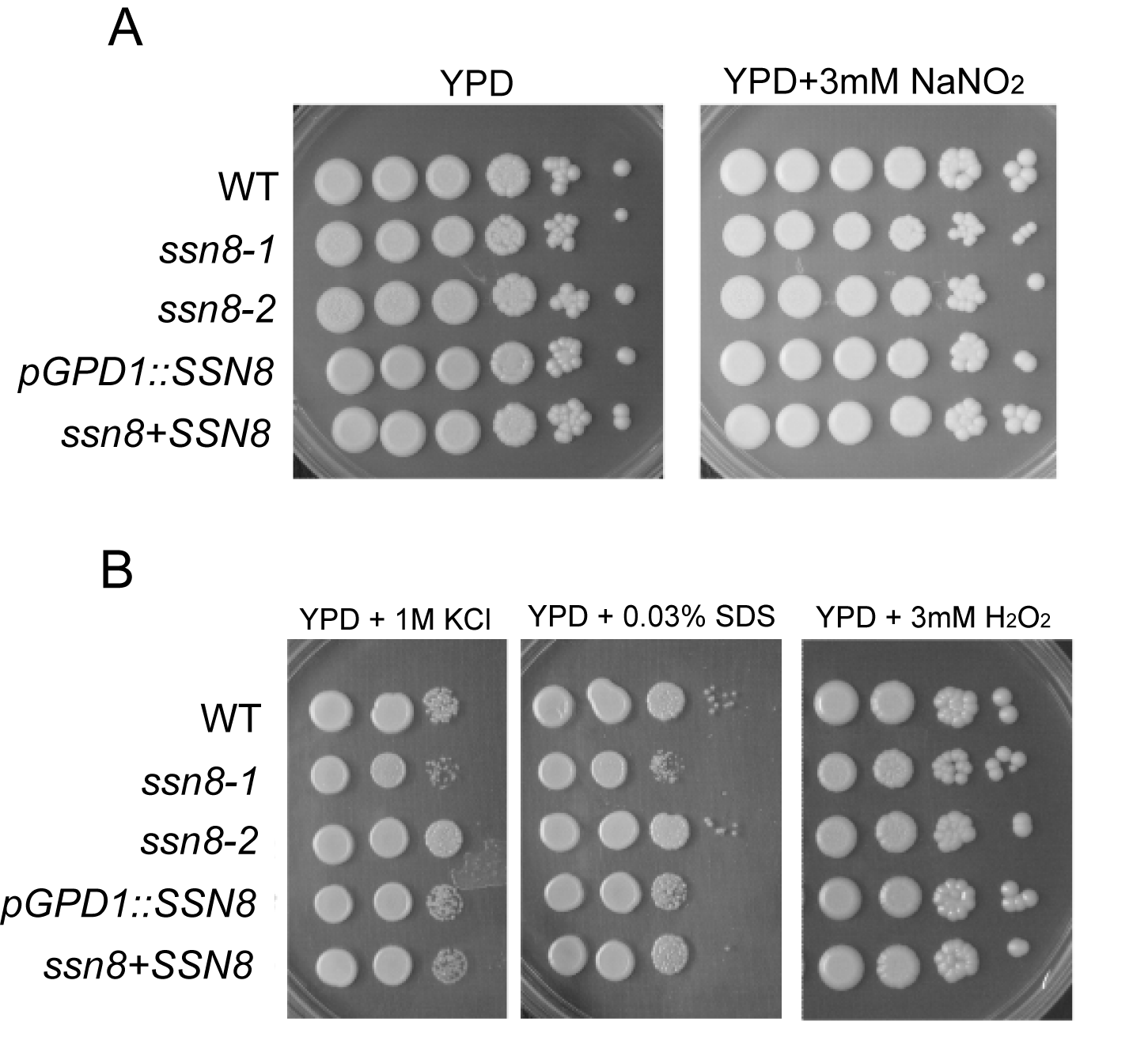

Supplement: Figure S6 — C. neoformans ssn8 mutant strains exhibit no growth defect under different stress conditions. The 10-fold diluted cells were spotted on YPD medium or YPD media containing different stress reagents, including 3 mM NaNO2 (A), 1 M KCl, 0.03% SDS, and 3 mM H2O2 (B). (TIF) [file pone.0019162.s006.tif]

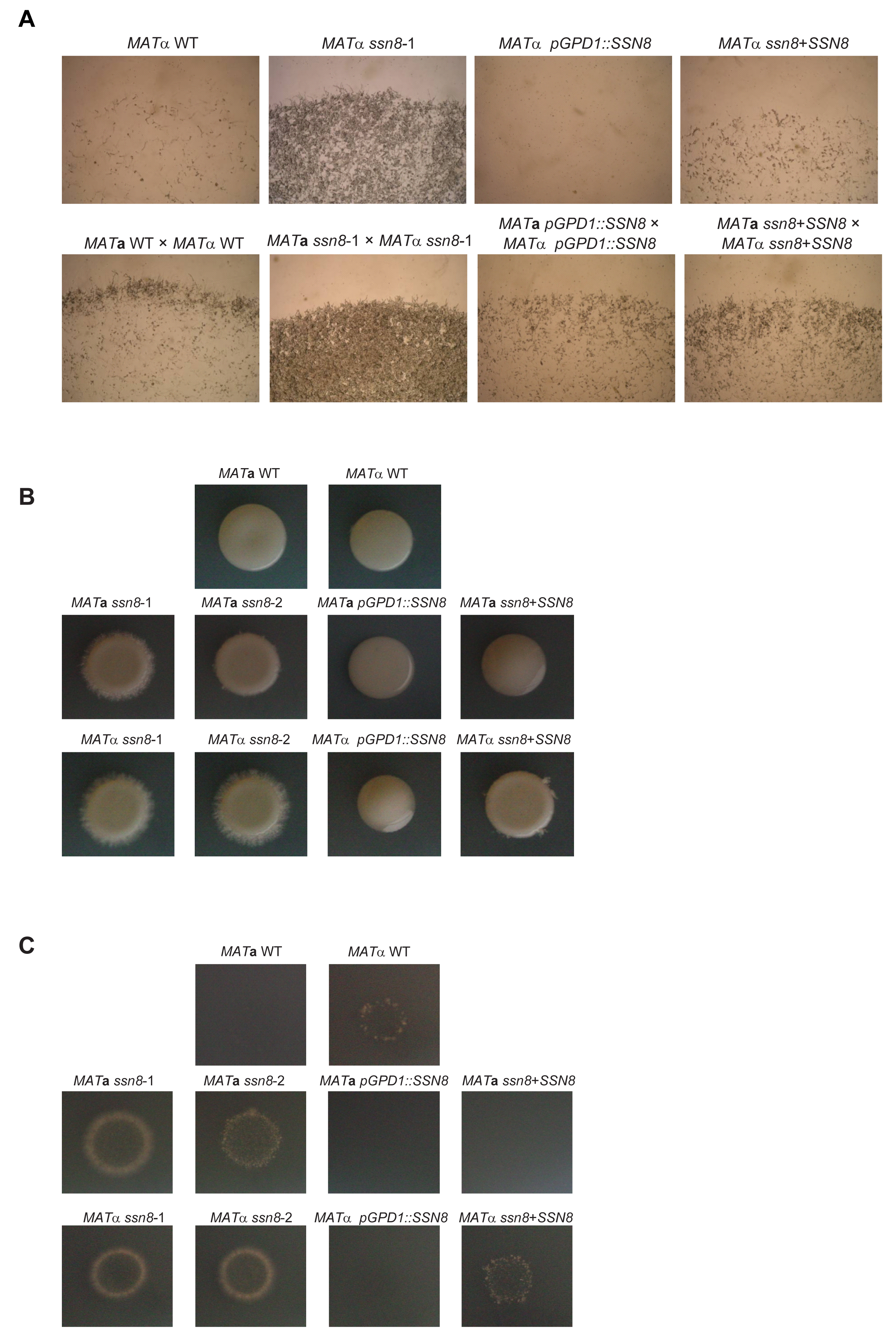

Supplement: Figure S7 — Deletion of SSN8 induces invasive growth on different media. (A) The ssn8 mutant strains displayed severe invasive growth on V8 medium at 26°C under light condition after 24 h. Colonies were washed off by sterile water and photos were taken. (B) C. neoformans strains as indicated were incubated on filament agar at 26°C for 9 days. The margins of the ssn8 mutant colonies showed more abundant hyphae than those of the wild-type, reconstituted and overexpression strains. (C) The 9-day fruiting colonies on filament agar were washed by sterile water and photos were taken. The ssn8 mutants showed more severe invasive growth than the wild-type strains. (TIF) [file pone.0019162.s007.tif]

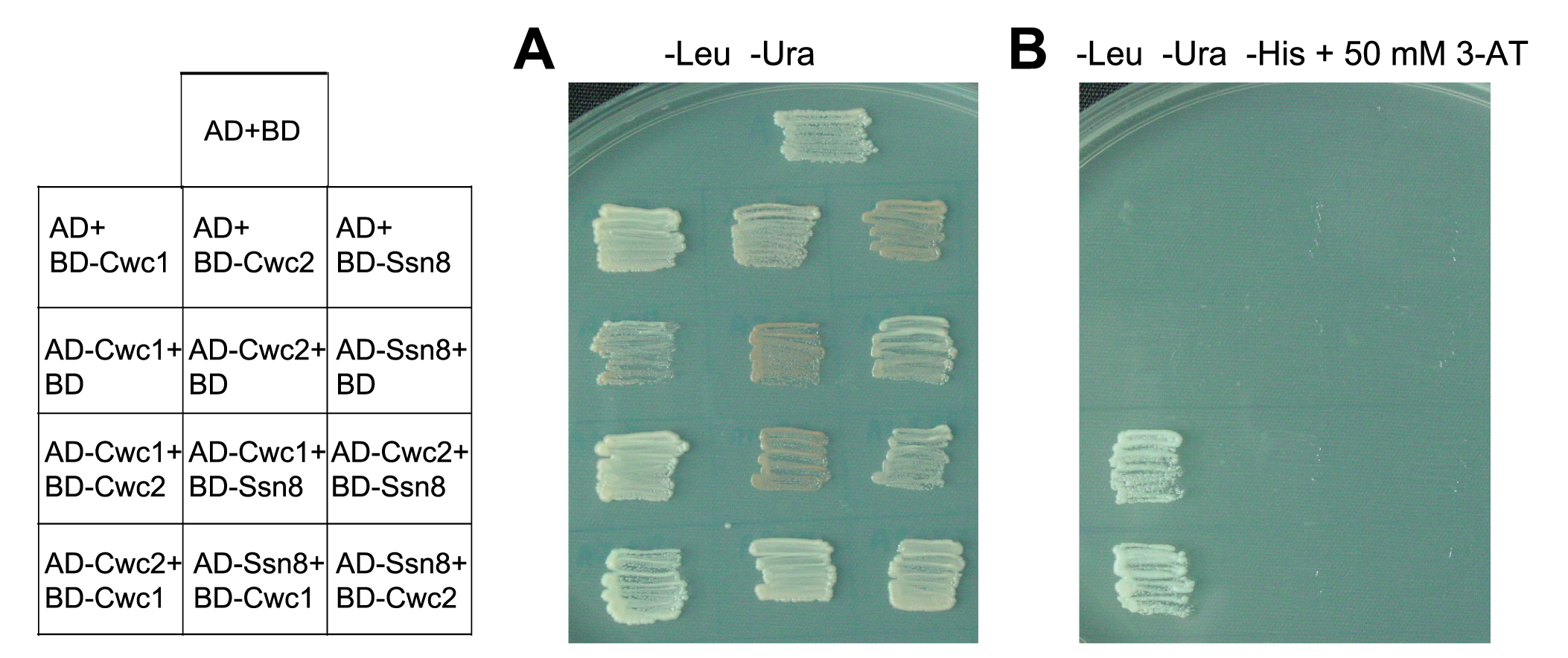

Supplement: Figure S8 — C. neoformans Ssn8 does not directly interact with the Cwc1 or Cwc2 protein. The coding sequences of SSN8, CWC1 and CWC2 were cloned into pGAD-C1 or pGBDU-C1 and cotransformed into S. cerevisiae strain PJ69-4A to test physical interaction. Transformants were streaked on SD-ura-leu medium (A) and SD-ura-leu-his+50 mM 3-AT medium (B) and positive interaction was detected only between Cwc1 and Cwc2. (TIF) [file pone.0019162.s008.tif]
