# Supplementary material for: Cryptococcus neoformans Mediator Protein Ssn8 Negatively Regulates Diverse Physiological Processes and Is Required for Virulence
Source: PLoS One. 2011 Apr 29;6(4):e19162. doi: 10.1371/journal.pone.0019162 (PMC3084776; doi:10.1371/journal.pone.0019162)
Supplement: Materials and Methods S1 — (DOC) [file pone.0019162.s009.doc]

**Supporting Information-Materials and Methods**

**Cell Growth Assay**

Strains subjected to growth assay were first grown on YPD agar for 2 days. A single colony was inoculated into 5 ml YPD liquid medium and incubated at 30^o^C for overnight. Cells were collected by centrifugation, washed with sterile water, and adjusted to a density of 4.8×10^7^ cells/ml. Ten-fold serial dilutions for each tested strain were conducted, and 10 μl of cell suspension from each dilution was then spotted onto YPD, FA, YNB and YNB, each with a different carbon sources (0.2% glucose/2% glucose/2% sucrose/2% ethanol/2% sodium acetate/2% glycerol/2% galactose), and onto YPD medium, each supplemented with a different stress reagents (0.5% Congo Red/1 M KCl/0.75 M NaCl/0.1% SDS/0.5 mg/ml caffeine/1.5 M calcofluor white/3 mM NaNO_2_/ 3 mM H_2_O_2_). These cultures were incubated at 30^o^C in the dark for 2-5 days and pictures were taken by a digital camera (Sony DSC-S85).

**Invasive Growth Assay**

Strains subjected to invasive growth assay was first grown overnight in 5 ml YPD liquid medium at 30^o^C. Five microliters of cell suspension was spotted on YPD, V8 and filament agar medium and incubated at 30^o^C for over 10 days. Colony was then repeatedly washed with sterile water using a bent glass rod to remove excess yeast cells from the surface of the agar. Cells remained on the plate were examined under a microscope (Olympus BX41) and photographed.
