# Supplementary material for: Cryptococcus neoformans Mediator Protein Ssn8 Negatively Regulates Diverse Physiological Processes and Is Required for Virulence
Source: PLoS One. 2011 Apr 29;6(4):e19162. doi: 10.1371/journal.pone.0019162 (PMC3084776; doi:10.1371/journal.pone.0019162)
Supplement: Table S1 — The relative expression levels of C. neoformans genes in the wild-type and SSN8 -related strains in YPD liquid medium. (DOC) [file pone.0019162.s010.doc]

**Supporting Information- Table S1**

**Table S1. The relative expression levels of *C. neoformans* genes in the wild-type and *SSN8*-related strains in YPD liquid medium**

| Strain  Gene | WT | *ssn8*-1 | *ssn8*-2 | *ssn8*-3 | *pGPD1::*  *SSN8* | *ssn8 +*  *SSN8* |
| --- | --- | --- | --- | --- | --- | --- |
| *SSN8* | 1 | undetermined | undetermined | undetermined | 48.75 ± 14.08 | 0.98 ± 0.11 |
| *MF*α | 1 | 5.06 ± 0.69 | 3.26 ± 0.62 | 2.51 ± 0.38 | 0.72 ± 0.29 | 1.30 ± 0.10 |
| *SXI1*α | 1 | 5.40 ± 1.41 | 6.50 ± 1.15 | 5.52 ± 0.49 | 0.79 ± 0.05 | 1.65 ± 0.10 |
| *GPB1* | 1 | 4.06 ± 0.25 | 4.00 ± 1.00 | 3.29 ± 1.25 | 0.94 ± 0.14 | 1.80 ± 0.31 |
| *STE11* | 1 | 3.09 ± 1.10 | 3.89 ± 0.72 | 2.43 ± 0.55 | 0.92 ± 0.10 | 1.39 ± 0.21 |
| *STE7* | 1 | 4.03 ± 2.17 | 2.39 ± 0.68 | 2.32 ± 0.33 | 0.96 ± 0.08 | 1.28 ± 0.11 |
| *STE12*α | 1 | 2.40 ± 0.89 | 3.15 ± 0.17 | 2.18 ± 0.35 | 0.95 ± 0.06 | 1.39 ± 0.12 |
| *MAT2* | 1 | 1.42 ± 0.15 | 2.08 ± 0.14 | 1.65 ± 0.26 | 1.55 ± 0.42 | 0.61 ± 0.09 |
| *ZNF2* | 1 | 2.81 ± 0.15 | 3.71 ± 0.18 | 2.64 ± 0.29 | 0.39 ± 0.17 | 1.01 ± 0.18 |
| *GPA2* | 1 | 9.09 ± 1.14 | 9.02 ± 1.63 | 6.07 ± 1.55 | 1.70 ± 0.86 | 087 ± 0.11 |
| *CPR2* | 1 | 52.67 ±18.11 | 31.49 ± 0.67 | 30.17 ± 1.67 | 0.41 ± 0.07 | 0.95 ± 0.08 |
| *CRK1* | 1 | 1.81 ± 0.93 | 2.60 ± 0.40 | 2.34 ± 0.17 | 0.61 ± 0.11 | 1.07 ± 0.23 |
| *SNF1* | 1 | 1.07 ± 0.03 | 1.58 ± 0.19 | 1.60 ± 0.31 | 0.31 ± 0.25 | 0.84 ± 0.41 |
| *HXT* | 1 | 3.60 ± 0.68 | 8.20 ± 1.04 | 8.80 ± 0.73 | 0.94 ± 0.05 | 0.95 ± 0.18 |
