# Supplementary material for: Cryptococcus neoformans Mediator Protein Ssn8 Negatively Regulates Diverse Physiological Processes and Is Required for Virulence
Source: PLoS One. 2011 Apr 29;6(4):e19162. doi: 10.1371/journal.pone.0019162 (PMC3084776; doi:10.1371/journal.pone.0019162)
Supplement: Table S2 — Primers used in this study. (DOC) [file pone.0019162.s011.doc]

**Supporting Information- Table S2**

**Table S2**. Primers used in this study

| Primer name | Sequence (5’ to 3’) |
| --- | --- |
| WC445 | CGG GAT CCA TGT CTT CCA ACT TCT ATA CCT CCT |
| WC446 | TCC CCC GGG CCA CTG CCA GAT ACT TCT CCG AA |
| WC458 | CAG AGC CAG ATT GAC CCC AA |
| WC466 | CCA GCT CTC AAC GCA TTT ACA AGC A |
| WC467 | CGG GAT CCT CTT TGG TCA ATT GGG CAG ATG G |
| WC468 | CGG GAT CCA TCG CAT GCG TAT ATG TAC ACT |
| WC469 | CGT TAC TGG TCG AGA CGT TCA GTT |
| WC530 | GCT CTA GAA ACA GCT GCT TGC ATA CGG TC |
| WC636 | GCG GAT CCC TAT CTT GCG CGC TTT TTA CCC GCT ACA TTG |
| WC536 | GGA ATT CCA TCA TCA CCA TCA CCA CAT GTC CCT CCT CGC CGA GTC T |
| WC537 | GGA ATT CTC AAG ATT GCT TAT CTT TTT TGG GA |
| WC341 | CGG GAT CCA TGT CAA CAA ACC TCA CTT CTC CGC A |
| WC342 | GCG GTG GCG GCC GCA CAT ATC AAA ATC CAA TAC CCC CA |
| WC515 | CGG GAT CCA TGG CAA CAA TTC CAG GCG GA |
| WC854 | AAC TGC AGT TAA ATT CTC GCC TTC |
| WC740 | GGA ATT CAT GCT TTC GAC CTG TGA TAG CTC T |
| WC741 | CGG GAT CCT TAA CAC GCT AGG CGC GGC TTG GT |
| WC511 | ATA TCA AGC TTC CAT AAA CTG CAC ACC AGC A |
| WC512 | GCT CTA GAG TTA CCC ACG CAC TTC CAT T |
| WC513 | CGG GAT CCG TTC AAC CAC GTC GGC TTT |
| WC514 | CTT GTC CAA CCT GCG ATC GA |
| WC516 | TCC CCC GGG TAT GCG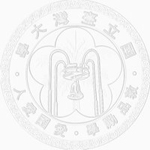 CTC TCT CAC TCT |
| Quantitative real time PCR primer | |
| WC503 | TGG AAG TGG ACG AGG G |
| WC504 | CGG TCG CTG AGT GGA A |
| WC531 | TGG TCA CCC CTT GTC ATC G |
| WC532 | TTC CGC ATC AGG ATC AGT CAT |
| WC572 | CTC GCT CAT TAG ACA GCA ACT CA |
| WC573 | GAA GAT GGC AGT GAA GGC GT |
| WC566 | TGA ACA GGG TGG AGA AAG GTA AG |
| WC567 | AGT GAA ACG GTA TTT GAA GGC G |
| WC576 | TCA GAG CAT TGG TCT TTC GTT TT |
| WC577 | TAA TAC AGC CAT TGC GGA ACA A |
| WC590 | CAT ACG GGC GAT GTG ATG AG |
| WC591 | CGG TGG CAA AGG CGT C |
| WC623 | AGG AGG CGG TTG CAT TTG T |
| WC624 | TTT GAG GTT GGT TAT CCA CGA AG |
| WC629 | GGA GCG TGA GAT TGA GTT GCT |
| WC630 | AAA TGC GCC GTA GTT GTT GAG |
| WC745 | CGG AAC GAC GAA AAT GGC T |
| WC746 | CAT GGC TTC TTG CAT ACG ATT G |
| WC792 | CCC CAT TTT GAT TGC CGT |
| WC793 | CAT CAG AGG CAA TAA GCG AAA G |
| WC796 | TAC GAG GAC GAG GAT TCA TCT G |
| WC797 | GCA CCA TTT TGC TGC ACA TC |
| WC798 | GGA AGC AAA AGG AGA CCA CCA C |
| WC799 | ATG TTA CGC AAC TCA GAA CCG G |
| WC800 | AGA TAT TGC TATACCCGCCACC |
| WC801 | CCA TGT AAG TAA TTT GCC CGC G |
| WC875 | CCA GAT ATC AGA GCG GTG TAC G |
| WC876 | TTT TCG GCC TTC CTC TTA GGT |
| WC877 | GAT GCT GCC GCT TGA AAT G |
| WC878 | TCG CGA GAC ATA GGC GTA TTC |
